# An intelligent responsive macrophage cell membrane-camouflaged mesoporous silicon nanorod drug delivery system for precise targeted therapy of tumors

**DOI:** 10.1186/s12951-021-01082-1

**Published:** 2021-10-24

**Authors:** Minghua Li, Xiaolong Gao, Chao Lin, Aijun Shen, Jing Luo, Qiongqiong Ji, Jiaqi Wu, Peijun Wang

**Affiliations:** 1 Department of Radiology, Tongji Hospital, School of Medicine, Tongji University, Shanghai, 200065 People’s Republic of China; 2 Department of Radiology, Luodian Hospital, Baoshan District, Shanghai, 201908 People’s Republic of China; 3 Department of Radiology, Luodian Hospital, Shanghai University, Shanghai, 200444 People’s Republic of China; 4 Institute for Translational Medicine, Shanghai East Hospital, Institute for Biomedical Engineering and Nanoscience, School of Medicine, Tongji University, Shanghai, 200092 People’s Republic of China

**Keywords:** Mesoporous silicon nanorods, Macrophage cell membrane, Drug delivery, Precision tumor therapy

## Abstract

Macrophage cell membrane-camouflaged nanocarriers can effectively reduce immune cell clearance and actively target tumors. In this study, a macrophage cell membrane-camouflaged mesoporous silica nanorod (MSNR)-based antitumor drug carrier equipped with a cationic polymer layer was developed. As drug carriers, these MSNRs were loaded with the thermosensitive phase change material L-menthol (LM), the chemotherapy drug doxorubicin (DOX) and the fluorescent molecule indocyanine green (ICG). The rod-like shape of the MSNRs was shown to enhance the penetration of the drug carriers to tumors. In the weakly acidic tumor microenvironment, the cationic polymer exhibited a proton sponge effect to trigger macrophage cell membrane coating detachment, promoting tumor cell uptake. Following nanocarrier uptake, ICG is heated by near-infrared (NIR) irradiation to make LM undergo a phase transition to release DOX and generate a synergistic effect of thermochemotherapy which kills tumor cells and inhibits tumor growth together with reactive oxygen species (ROS) produced by ICG. Overall, this nanohybrid drug delivery system demonstrates an intelligent cascade response, leads to tissue-cell specific targeting and improves drug release accuracy, thus proving to be an effective cancer therapy.

**Supplementary Information:**

The online version contains supplementary material available at 10.1186/s12951-021-01082-1.

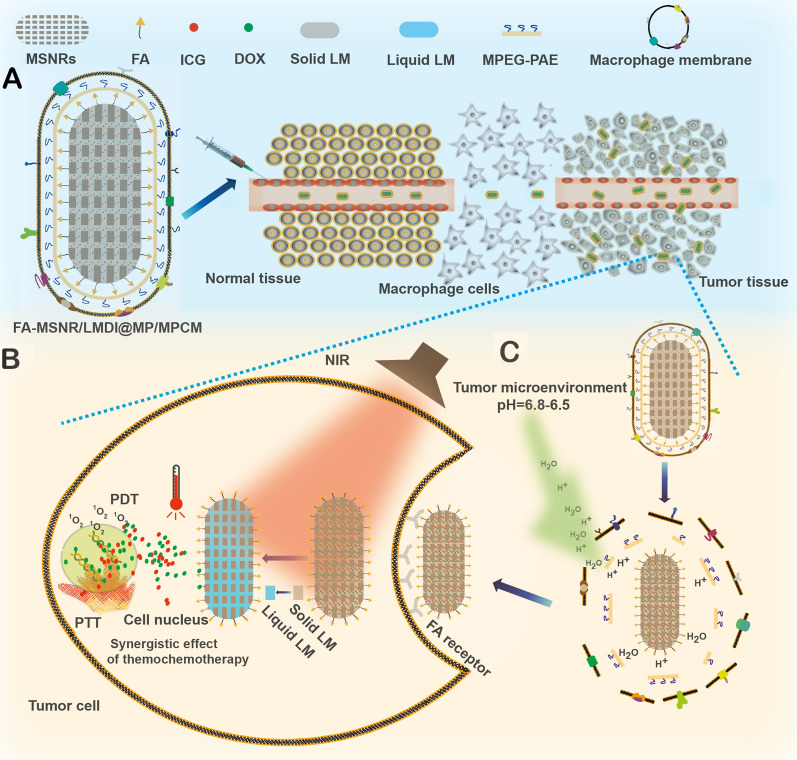

## Background

While nanomaterials are being developed as drug carriers, some factors, including biocompatibility, immune evasion, tumor targeting, penetration and cellular uptake, must be considered to optimize the antitumor effect. Owing to their high surface area, stability, uniform particle size, pore diameter, facile surface functionalization, and biocompatibility, mesoporous silica nanoparticles (MSNs) have been widely utilized in research on anticancer drug carriers [1–4]. Currently, the most commonly used MSNs are spherical in shape with a diameter of approximately 100–200 nm, but these parameters make penetration into the deep layer of tumor tissue difficult. However, alternative mesoporous silica nanorods (MSNRs) have been shown to provide improved tumor penetration and a higher cell uptake rate than spherical MSNs due to their rod-shaped morphology [5–10].

Due to the lack of high selectivity and specificity, the aggregation effect of nanocarriers without active targeting on tumor tissues is low [11–14]. Therefore, nanocarriers with specific modifications that enable binding to tumor cell surface receptors or antigens are desirable for site-specific targeting [15–18]. The hydrophobic and electrical properties of these targeted groups on nanocarriers may reduce their time in circulation. Moreover, as exogenous substances, nanocarriers are potentially at risk of reticuloendothelial system (RES) elimination, which would also significantly shorten their time in circulation and reduce the amount of drug reaching the tumor [19, 20].

Cell membrane biomimetic nanocarriers rely on the cell membrane to serve as the outer shell enclosing the nanoparticle. They combine the advantages of the cell membrane and nanoparticles to form a new type of biomimetic nanosystem [21–29]. Since mononuclear macrophage cells are immune cells, a nanocarrier cloaked in the macrophage cell membrane provides a camouflage effect and enables evading immunological surveillance. Moreover, mononuclear macrophage cells are abundant cells in the tumor microenvironment [30, 31] and can express a variety of proteins that bind to adhesion molecules on the surface of tumor cells. Hence, mononuclear macrophage cell membrane-encapsulated nanocarriers not only can avoid immune clearance and prolong the time in circulation but can also actively target the tumor tissue, thereby increasing the accumulation of drugs in the tumor. However, unfortunately, when macrophage cell membrane-encapsulated nanocarriers accumulate in the tumor tissue, the cell membrane becomes an obstacle prohibiting nanoparticle uptake by the tumor cells. Therefore, it is essential to identify a suitable cell membrane detachment strategy to increase tumor cell phagocytosis.

Methoxy poly(ethylene glycol)-poly(β-amino ester) (MPEG-PAE) is a cationic polymer with low cytotoxicity [32–34]. In comparison with healthy tissues (pH 7.4), the tumor microenvironment is weakly acidic (pH 6.5–6.8) [35, 36]. Inspired by the proton sponge effect [37, 38], cell membrane biomimetic nanocarriers that contain MPEG-PAE micelles, which can induce excessive H^+^ and water inflow in the tumor microenvironment to realize an internal balance of electric neutrality and ionic strength, cause MPEG-PAE micelles to swell [39, 40] and the cell membrane coating to burst and detach, thereby facilitating uptake of the released nanoparticles by tumor cells. Therefore, pH-sensitive MPEG-PAE could be used as an ideal cationic polymer for cell membrane biomimetic nanocarriers that can enable cell membrane detachment following exposure to the tumor microenvironment.

Indocyanine green (ICG) is a fluorescent molecule. ICG heats up and produces reactive oxygen species (ROS) with near-infrared (NIR) light irradiation. Thus, tumor cells are destroyed by both heat and ROS, combining photothermal therapy (PTT) and photodynamic therapy (PDT) [41–43]. Additionally, many chemotherapeutics can be more lethal to tumor cells in an environment with temperatures above body temperature. Compared with chemotherapy alone, thermochemotherapy provides a more robust therapeutic effect [44–49].

**Fig. 1:**
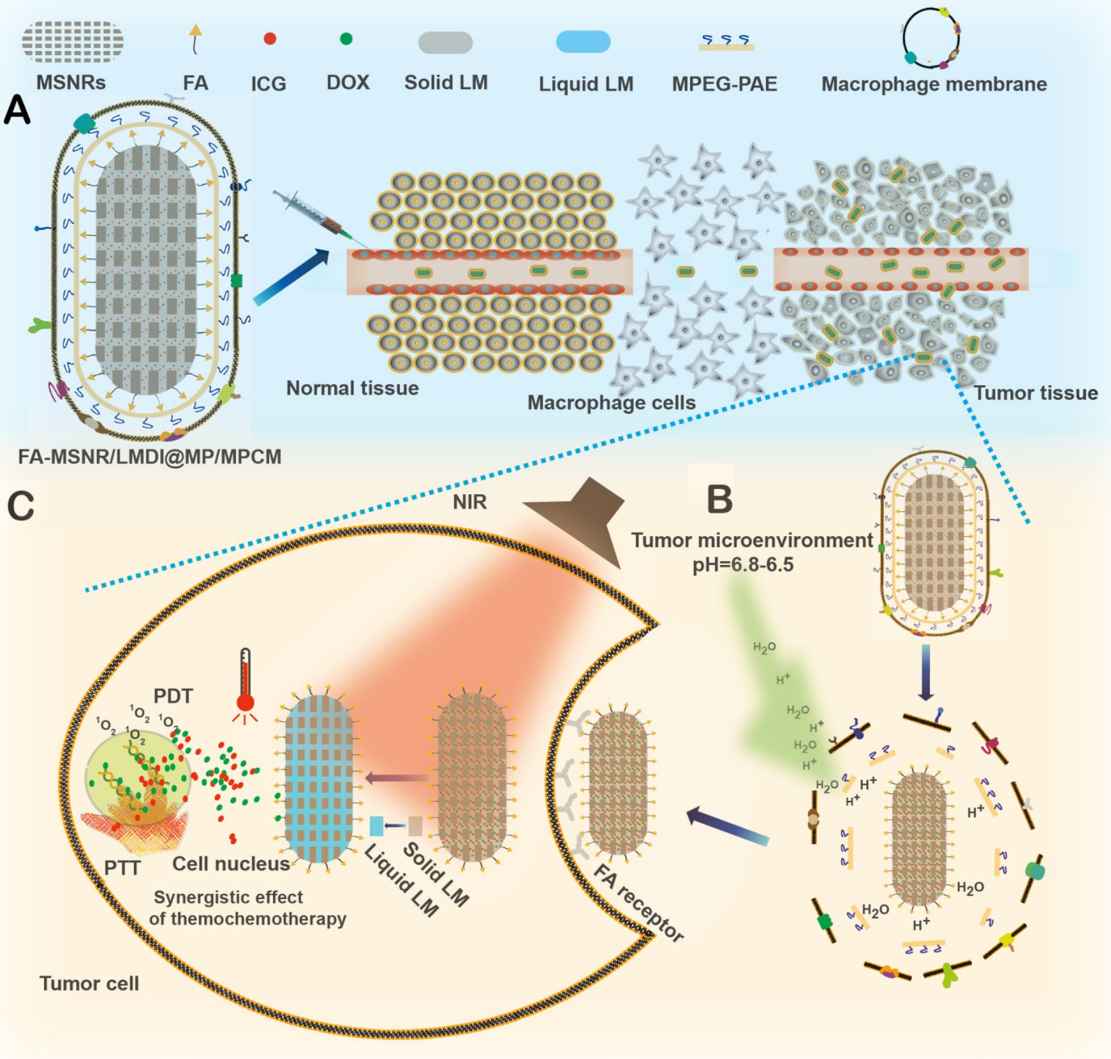
Schematics showing the structure of the intelligent responsive macrophage cell membrane-camouflaged MSNR drug delivery system for accumulation, drug release and treatment in tumor tissue. **A** Macrophage cell membrane coating mediated immune evasion, active tumor targeting, and deep tumor penetration. **B** Weakly acidic tumor microenvironment mediated membrane detachment to promote uptake of nanocarriers by tumor cells. **C** NIR mediated enhanced synergism of PTT, PDT and chemotherapy

**Fig. 2:**
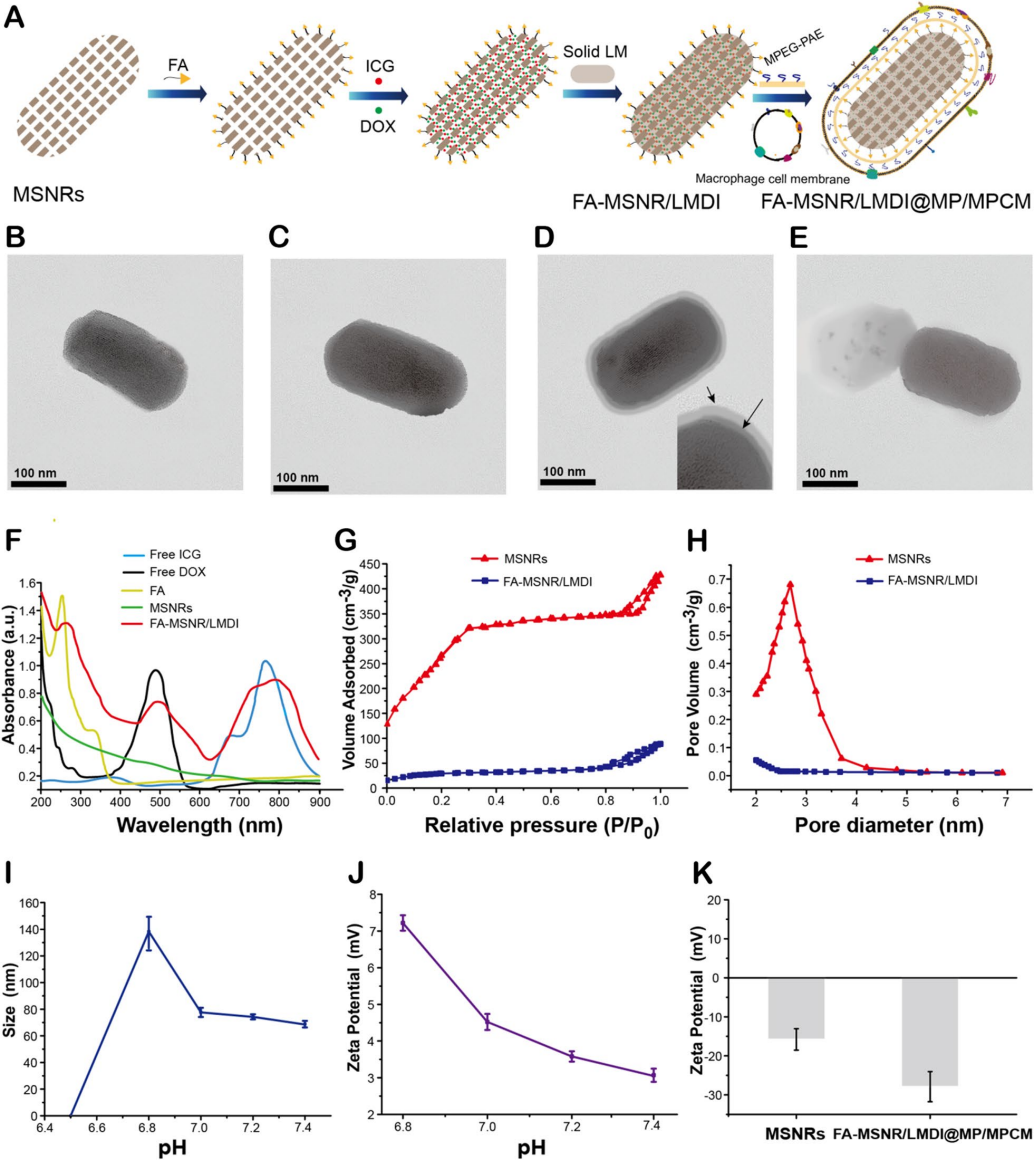
Synthesis and characteristics of an intelligent responsive macrophage cell membrane-camouflaged MSNR drug delivery system. **A** Schematics showing the synthesis of FA-MSNR/LMDI@MP/MPCM. **B** TEM image of MSNRs. **C** TEM image of FA-MSNR/LMDI. **D** TEM image of FA-MSNR/LMDI@MP/MPCM. The inset image: macrophage cell membrane (indicated by the short arrows); MPEG-PAE (indicated by the long arrows). **E** TEM image of membrane detachment of FA-MSNR/LMDI@MP/MPCM in a weakly acidic environment. **F** UV–Vis spectroscopy of free DOX, free ICG, FA, MSNRs and FA-MSNR/LMDI. **G** Nitrogen adsorption–desorption isotherms of MSNRs and FA-MSNR/LMDI. **H** Pore size distribution plots of MSNRs and FA-MSNR/LMDI. **I** Changes in particle sizes of MPEG-PAE at various pH values. **J** Changes in zeta potentials of MPEG-PAE at various pH values. K Zeta potentials of MSNR and FA-MSNR/LMDI@MP/MPCM

On the basis of the above information, an intelligent responsive MSNR drug delivery system coated with macrophage cell membranes was designed. In this system, we used MSNR surface-modified folic acid (FA) as a carrier, loaded with doxorubicin (DOX), ICG and L-menthol (LM) which are thermosensitive phase change materials, as gating molecules. We then covered MSNRs with the pH-sensitive cationic polymer MPEG-PAE, and finally encapsulated the macrophage cell membrane (Fig. 2A). This drug delivery system combines the advantages of deep tumor penetration of MSNRs, tumor targeting and immune evasion of macrophage cell membrane coatings. When it enters the weakly acidic tumor microenvironment, the MPEG-PAE swells and ruptures, subsequently enabling MSNRs to escape from the membrane coating. Furthermore, the exposed MSNR surface-modified FA can specifically bind to the highly expressed FA receptor of tumor cells to improve phagocytosis. Moreover, ICG in the drug delivery system is heated by NIR in tumor cells, which triggers LM to undergo a phase transition and release DOX, and through the synchronization of photothermal-chemotherapy, DOX and the ROS produced by ICG kill tumor cells (Fig. 1).

## Methods

### Synthesis of FA-MSNR/LMDI@MP/MPCM

#### Preparation of MSNRs

CTAB was dissolved in H_2_O (70 mL), and NH_3_.H_2_O was added with stirring for 1 h. TEOS was then added with vigorous stirring for 4 h at room temperature. The molar ratio of short rod- and sphere-shaped particles was 200 (CTAB):1000 (H_2_O):20 (NH_3_.H_2_O):1.4 (TEOS) and 100 (CTAB):1000(H_2_O):10 (NH_3_.H_2_O):0.7 (TEOS), respectively. MSNs of different shapes were collected after centrifugation at 15,000*g* for 20 min, washed with and redispersed with ethanol and deionized water. Surfactant templates were removed by gradient calcination (100 W, 500 °C) for 5 h.

#### Preparation of FA-MSNRs

The *N*-hydroxysuccinimide ester of folic acid (NHS-folate) was prepared by esterification of folic acid (1 mmol) with NHS (1 mmol) in dry dimethylsulfoxide (DMSO, 0.4 mL) solution of EDC (2 mmol) and HOBT (1 mmol). Then, NHS folate was added to the MSNR-NH_2_ suspension and stirred in a nitrogen atmosphere for 72 h at room temperature. The mixture was washed with deionized water to produce FA-MSNRs.

Preparation of FA-MSNR/LMDI. FA-MSNRs (10 mg), DOX (8 mg), ICG (1.5 mg), and LM (4 mg) were first dispersed in ethanol (10 mL). Then, the mixture was heated to 50 °C and stirred for 10 h to completely evaporate the ethanol. After that, hot water (15 mL) was added, and the mixture was immediately centrifuged at 50 °C. Subsequently, the product was collected through centrifugation.

#### Preparation of FA-MSNR/LMDI@MP

FA-MSNR/LMDI (10 mL) was added to 30 mg of Pluronic F-127 and 2 mL of MPEG-PAE (20 mg) trichloromethane solution. The solution was stirred rapidly at room temperature for 12 h, centrifuged at 10,000 rpm for 30 min, and washed twice with ultrapure water. FA-MSNR/LMDI@MP was stored in 10 mL ultrapure water.

#### Preparation of FA-MSNR/LMDI@MP/MPCM

As-prepared MPCM ghosts were dispersed in PBS and treated with ultrasonication. MPCM was mechanically extruded through a Millipore membrane to form MPCM vesicles by a mini-extruder. FA-MSNR/LMDI@MP/MPCM was prepared by the mixture of FA-MSNR/LMDI@MP and MPCM vesicles through a polycarbonate membrane for extrusions.

### Nanocarriers penetration study

For construction of tumor spheroids, 4000 4T1 cells in 400 µL of DMEM were cultured in 100 µL of 4% agarose-pretreated 48-well plates and maintained for 7 days, and tumor spheroids were obtained.

For the in vitro penetration study, the tumor spheroids were incubated with the four types of nanocarriers in different media (pH 7.4 and pH 6.5) and then washed, fixed and transferred to a flat glass-bottom petri dish for observation under a fluorescence microscope (Ti-S, Nikon, Japan).

For the in vivo penetration study, tumor-bearing nude mice injected with four types of nanocarriers were sacrificed 24 h after treatment. The tumor tissue was cut into 10 μm slices, which were prepared and stained with CD34 antibody and DAPI to visualize vessels and nuclei. The slices were then observed and photographed under a fluorescence microscope to observe the nanocarrier distribution in the tumor region.

### In vivo distribution of FA-MSNR/LMDI@MP/MPCM

After individual tail vein injection of the four types of nanocarriers into tumor-bearing nude mice, real-time fluorescence imaging was performed to observe the distribution changes of the nanocarriers with time. The excitation wavelength of the filter was 780 nm, and the emission wavelength was 840 nm.

Tumor-bearing nude mice injected with four types of nanocarriers were sacrificed after 24 h to harvest their tumors, hearts, livers, spleens, lungs and kidneys. The nanocarrier distribution of the tumors and each organ was observed by live fluorescence imaging (Nightowl LB981, Berthold, Germany), and the relative intensity of ICG fluorescence was quantitated and compared.

### In vitro cytotoxicity

Mice breast cancer cells (4T1), human hepatocytes (L-02) and human embryonic kidney cells (293T) were seeded in 96-well cell culture plates, with each well containing 1 × l0^4^ cells, and then cultured for 24 h. Then, L-02 cells and 293Tcells were treated with different MSNRs concentrations in medium for 24 h, and 4T1 cells were treated with MSNRs, FA-MSNR/LMDI@MP/MPCM at pH 7.4, FA-MSNR/LMDI@MP/MPCM at pH 6.4, FA-MSNR/LMDI@MP/MPCM + NIR (808 nm, W cm^−2^) at pH 6.5 and free DOX with different MSNRs or DOX concentrations in medium for 24 h. In the process of incubation, the cells were exposed to NIR radiation for 2 min after 4 and 8 h. Cell viability was calculated by standard MTT assay.

4T1 cells with different treatments (MSNRs, FA-MSNR/LMDI@MP/MPCM at pH 7.4, FA-MSNR/LMDI@MP/MPCM at pH 6.4, and FA-MSNR/LMDI@MP/MPCM + NIR (808 nm, 1.5 W cm^−2^) at pH 6.5 were stained with an AM/PI mixture, where AM was excited at 488 nm and PI was excited at 533 nm. The cells were observed through fluorescence microscopy to distinguish between dead and living cells.

Annexin V-FITC/PI staining and flow cytometry were used to detect 4T1 cell apoptosis. 4T1 cells were cultured at a density of 1 × 10^5^ cells mL^−1^. After treatment with trypsin, the cells with different treatments were stained with Annexin V-FITC/PI in the dark. Then, the cells were collected from each culture dish, and the fluorescence intensity was detected by flow cytometry, with the FL-1H channel detecting FITC at 488 nm and 530 nm. Untreated cells were used as the negative control.

### In vivo anticancer studies

To start tumor suppression experiments in vivo, we observed the nude mice bearing tumors until their tumor size reached a diameter of 1 cm. Next, we divided the nude mice into 4 groups (n = 4 in each group). Then, we anaesthetized the nude mice and administered different treatments intravenously (DOX dose of 1000 μg kg^−1^): (I) PBS; (II) free DOX; (III) FA-MSNR/LMDI@MP/MPCM and (IV) FA-MSNR/LMDI@MP/MPCM + NIR. Twenty-four hours after injecting the mice, we exposed certain groups to NIR irradiation (808 nm, 1.5 W cm^−2^), and sited them for 20 min. Animals were administered the corresponding treatments every 3 days.

The tumor sizes were monitored every 3 days for a total of 18 days. The tumor volume was calculated as follows: tumor volume V (mm^3^) = a (long diameter, mm) × b^2^ (short diameter, mm^2^)/2. The relative tumor volume = V/V_0_, where V_0_ was the tumor volume at the beginning of treatment, and V was the tumor volume after treatment. We also measured the body weights of the mice at the beginning and the end of treatment. The relative body weight = W/W_0_, where W_0_ was the body weight at the start of treatment.

## Results and discussion

### Synthesis and characterization of FA-MSNR/LMDI@MP/MPCM

MSNs were prepared via a condensation reaction using dilute tetraethyl orthosilicate (TEOS) and low surfactant conditions with NH_3_.H_2_O as a catalyst. The morphology of MSNs was controlled by the molar ratio of the reaction mixture [7, 8]. We adjusted the molar ratios of the mixture to 200 (CTAB): 1000 (H_2_O): 20 (NH_3_.H_2_O): 1.4 (TEOS) to synthesize MSNRs with a length of 180 nm and a width of 90 nm (aspect ratios (AR) = 2) as drug carriers. Additionally, spherical MSNs with a diameter of 100 nm were synthesized as controls (Additional file 1: Figure S1B, S2E). Examination by transmission electron microscopy (TEM) revealed the highly ordered mesoporous structure and porous morphology of MSNRs (Fig. 2B, Additional file 1: Figure S2A). We modified the targeting molecule FA on the surface of MSNRs and loaded LM, DOX and ICG in the mesoporous pores to construct FA-MSNR/LM.DOX.ICG (FA-MSNR/LMDI) (Fig. 2C, Additional file 1: Figure S2B). UV–Vis spectroscopy showed (Fig. 2F) that FA-MSNR/LMDI had characteristic absorption peaks of FA, DOX and ICG, thus confirming the modification of FA and encapsulation of DOX and ICG. Furthermore, the drug-loading content (DLC) of DOX was 11.2% and DLC of ICG was 15.7% as determined by UV–Vis spectroscopy.

LM is a thermosensitive phase transition material with high biocompatibility and phase transition temperature (Tm) of 43 °C [50, 51]. Since LM is a solid below 43 °C, it can be used as a gating molecule to encapsulate loads inside the mesoporous pores. When LM undergoes a phase transition into a solid–liquid phase above 43 °C, the gated channels are opened, and the loaded therapeutic agents are released from the pores [52]. Additionally, when the temperature is higher than 43 °C, tumor cells become sensitized to chemotherapeutic drugs, thereby achieving the synergistic effect of thermalchemotherapy. The specific surface area of the MSNRs was 1025.7 cm^2^ g^−1^, and the pore diameter was 2.5 nm. Owing to the blocking of mesoporous pores by solid LM and the encapsulated loads, the specific surface area of FA-MSNR/LMDI was greatly reduced to 108.3 cm^2^ g^−1^, and the pore diameter was close to zero (Fig. 2G, H).

Next, the FA-MSNR/LMDI surface was coated with the cationic polymer MPEG-PAE and wrapped with macrophage cell membrane (Additional file 1: Figure S1A) to form FA-MSNR/LMDI@MPEG-PAE/macrophage cell membrane (FA-MSNR/LMDI@MP/MPCM). MPEG-PAE consists of hydrophilic block (MPEG) and pH sensitive hydrophobic block (PAE). Its chemical structure was described in Additional file 1: Figure S3A. ^1^HNMR and GPC were used to confirm the chemical structure and molecular weight of the cationic polymer (Additional file 1: Figure S3B, C). MPEG-PAE contains a tertiary amine group in its skeleton, and it absorbs H^+^ and becomes protonated when in an acidic environment, which causes swelling [29], thereby breaking and detaching the macrophage cell membrane coating (Fig. 2E). As shown in Fig. 2I, J and Additional file 1: Figure S4, with a decreased pH in the range of pH 7.4 to 6.8, the zeta potential and dynamic light scattering (DLS) of MPEG-PAE gradually increased, while at pH 6.5, the polymer size decreased sharply, thus indicating complete dissolution of the polymer.

TEM images showed that the outer layer of FA-MSNR/LMDI@MP/MPCM was a macrophage cell membrane, and the next layer was MPEG-PAE. Then, the mesoporous structure of the internal MSNRs was unclear, and the electron density was increased (Fig. 2D, Additional file 1: Figure S2D). Furthermore, the zeta potentials of MSNRs and FA-MSNR/LMDI@MP/MPCM were − 15.8 ± 2.74 mV and − 27.9 ± 3.87 mV, respectively (Fig. 2K).

In addition to FA-MSNR/LMDI@MP/MPCM, we also prepared FA-MSNR/LMDI, which lacked MPEG-PAE and a macrophage cell membrane (Fig. 2C, Additional file 1: Figure S2B), FA-MSNR/LMDI@MPCM, which lacked MPEG-PAE (Additional file 1: Figures S1C, S2C), and FA-MSN/LMDI with spherical MSNs as the carrier, which lacked MPEG-PAE and a macrophage cell membrane (Additional file 1: Figures S1D, S2F). They were all examined to assess their immune evasion abilities, tumor-targeted phagocytic and penetration effects.

### Cell uptake and tumor penetration of nanocarriers

**Fig. 3:**
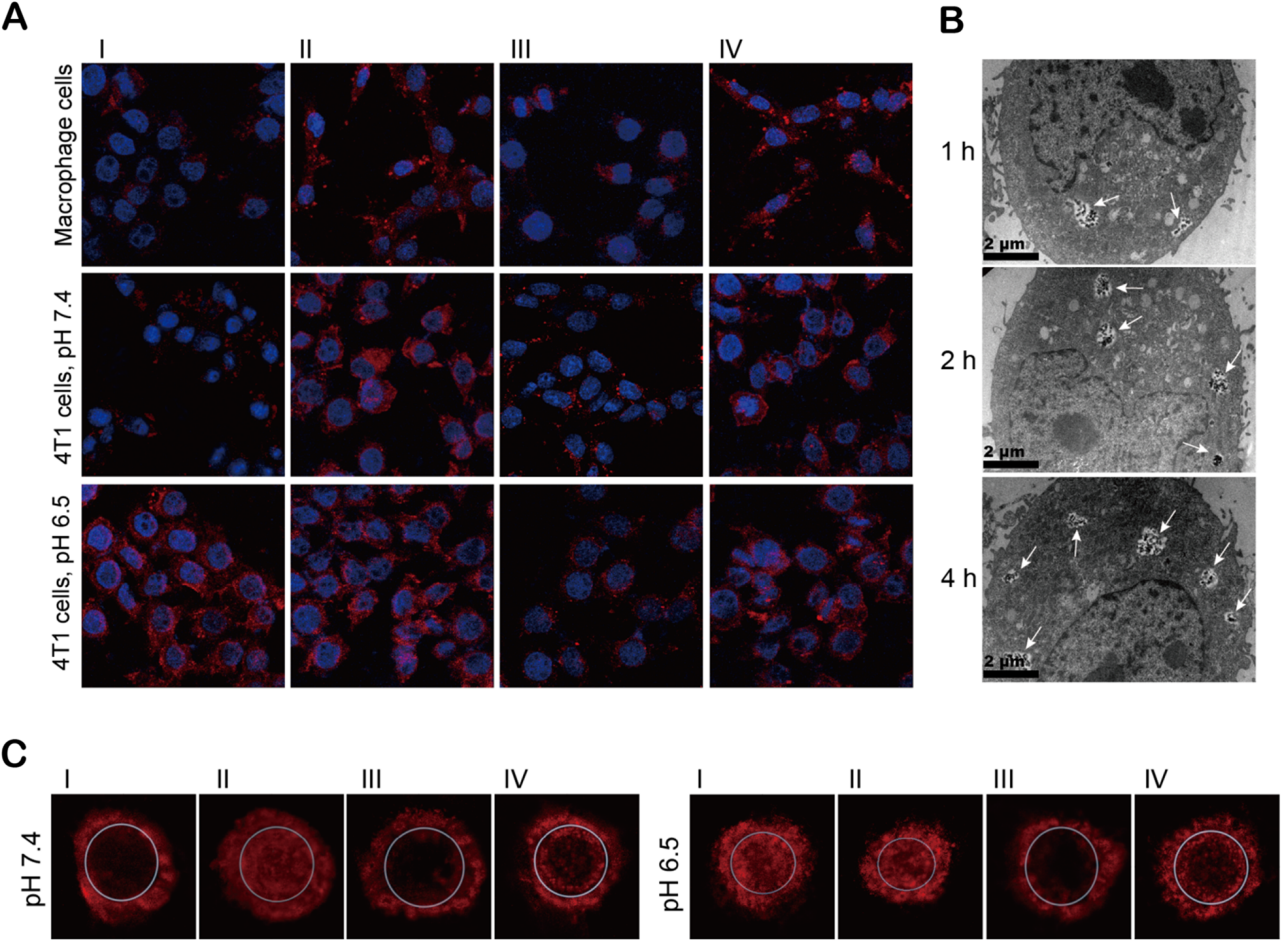
Cell uptake and tumor penetration of the four types of nanocarriers in vitro. **A** LSCM images showing the uptake of the four types of nanocarriers by 4T1 cells and macrophages. Blue: DAPI; red: nanocarriers. **B** TEM images of 4T1 cells incubated with FA-MSNR/LMDI@MP/MPCM (indicated by the arrows) for 1, 2 and 4 h. **C** LSCM images of 4T1 cell spheroids incubated with the four types of nanocarriers at pH 7.4 and pH 6.5. (I: FA-MSNR/LMDI@MP/MPCM; II: FA-MSNR/LMDI; III: FA-MSNR/LMDI@MPCM; IV: FA-MSN/LMDI)

Phagocytosis by macrophage cells in RES can accelerate the clearance of nanocarriers in vivo and reduce drug accumulation in tumor sites [17]. Therefore, the uptake of the four types of nanocarriers by macrophage cells was examined. The results showed that compared with that of FA-MSNR/LMDI and FA-MSN/LMDI, the uptake of FA-MSNR/LMDI@MP/MPCM and FA-MSNR/LMDI@MPCM by macrophage cells was significantly reduced (Fig. 3A and Additional file 1: Figure S5). This result indicated that the macrophage cell membrane on the surface of the nanocarriers successfully provided immune camouflage and enabled the evasion of macrophage phagocytosis.

Then, the uptake of the four types of nanocarriers by 4T1 breast cancer cells was evaluated. In the blood-mimicking environment (pH 7.4), the uptake of FA-MSNR/LMDI and FA-MSN/LMDI by 4T1 cells was more significant than that of FA-MSNR/LMDI@MP/MPCM and FA-MSNR/LMDI@MPCM due to the obstruction caused by intact macrophage cell membranes. However, in the tumor microenvironment-mimicking environment (pH 6.5), the uptake of FA-MSNR/LMDI@MP/MPCM significantly increased owing to the weakly acidic environment triggering macrophage cell membrane coating detachment, which then exposed the surface-modified FA and promoted the phagocytosis of tumor cells. Because the FA-MSNR/LMDI@MPCM did not have MPEG-PAE, the macrophage cell membrane coating was still wrapped in a weakly acidic environment, and therefore, tumor cell uptake was not obviously increased at pH 6.5 (Fig. 3A and Additional file 1: Figure S5). Additionally, FA-MSNR/LMDI@MP/MPCM particle uptake in 4T1 cells gradually increased with time (Fig. 3B).

**Fig. 4:**
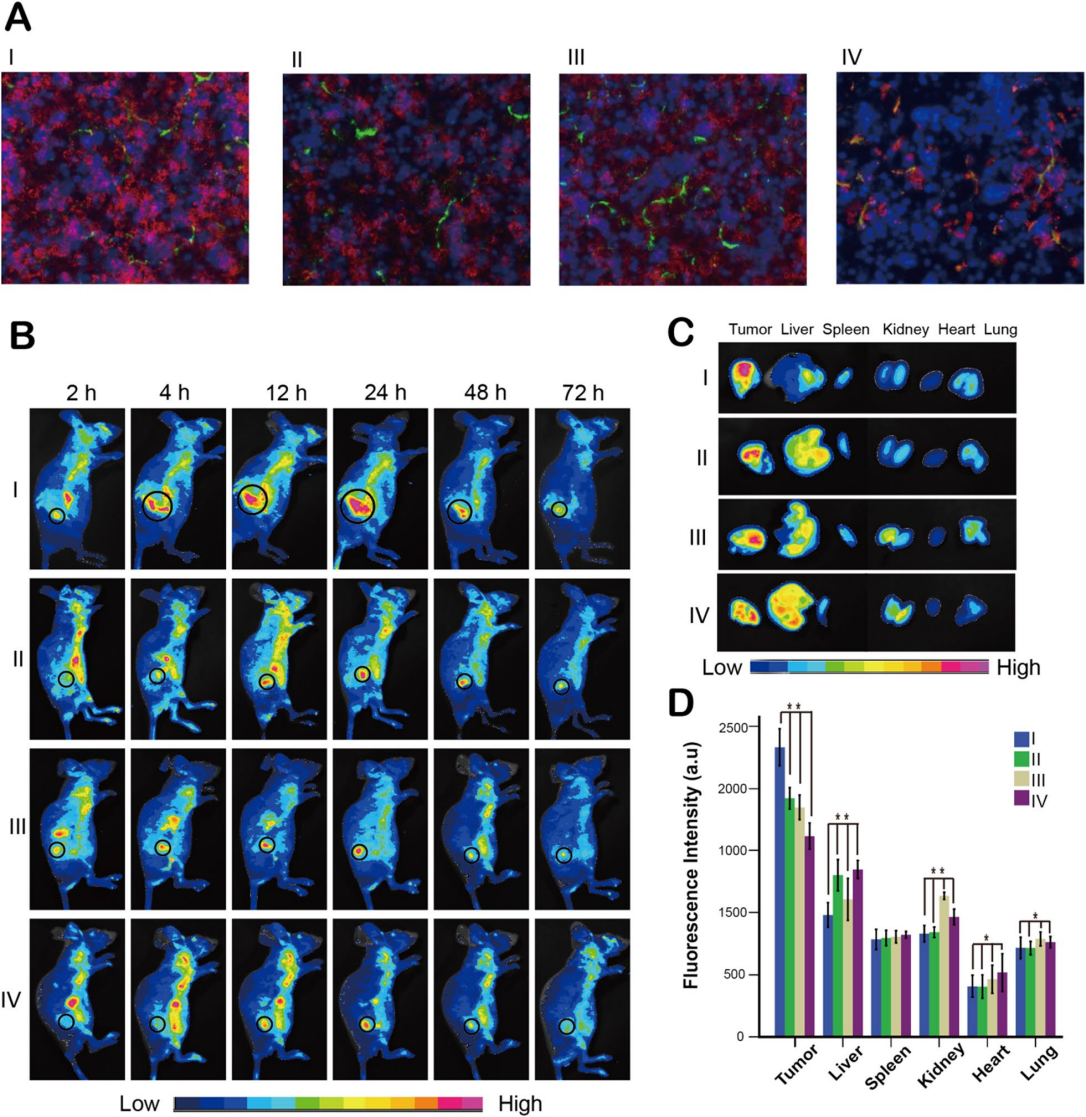
Tumor penetration and biodistribution of nanocarriers in vivo. **A** tumor sections of tumor-bearing nude mice 24 h after injection with the four types of nanocarriers. Blue: DAPI; green: CD34; red: nanocarriers. **B** In vivo real-time fluorescence images of tumor-bearing nude mice before and after injection with four types of nanocarriers. The black circles show the tumor areas. **C** Ex vivo organ fluorescence images of tumors and various organs 24 h postinjection. **D** Fluorescence intensities of various organs 24 h postinjection. *P < 0.05, **P < 0.01 was analyzed by one way analysis of variance (ANOVA). (I: FA-MSNR/LMDI@MP/MPCM; II: FA-MSNR/LMDI; III: FA-MSNR/LMDI@MPCM; IV: FA-MSN/LMDI)

When assessing the antitumor efficacy of a nanocarrier, deep tumor penetration is an important factor. Herein, 4T1 cell spheroids were built as in vitro tumor models to evaluate tumor penetration. Figure 3C revealed that, at pH 7.4, the fluorescence intensity of MSNR/LMDI@MP/MPCM and FA-MSNR/LMDI@MPCM within the 4T1 cell spheroids was weak owing to the obstruction of macrophage cell membranes; however, at pH 6.5, FA-MSNR/LMDI@MP/MPCM eliminated macrophage cell membranes and targeted tumor cells actively through exposed FA, and then, the fluorescence within the 4T1 cell spheroids was obviously enhanced. Furthermore, due to the limitation of size and morphology, FA-MSN/LMDI mainly gathered at the periphery of the 4T1 cell spheroids at both pH 6.5 and pH 7.4. Subsequently, tumor penetration was examined in vivo. After injecting the nanocarriers into tumor-bearing nude mice, DAPI and CD34 staining of tumor sections was utilized to visualize the tumor cells and vessels. The images showed that, due to its morphology, FA-MSN/LMDI was localized only to areas lining tumor vessels, while the other three types of nanocarriers that contained MSNRs were distributed within the tumor parenchyma away from the tumor vessels (Fig. 4A). Among them, FA-MSNR/LMDI@MP/MPCM showed the highest fluorescence intensity. Moreover, FA-MSNR/LMDI@MP/MPCM gradually moved from the vessels to the surrounding tumor tissues with time (Additional file 1: Figure S6).

### Pharmacokinetics and distribution of nanocarriers in vivo

First, the in vivo pharmacokinetics of free DOX, FA-MSNR/LMDI and FA-MSNR/LMDI@MP/MPCM were carried on (Additional file 1: Figure S7, Table S1). Compared with free DOX and FA-MSNR/LMDI, FA-MSNR/LMDI@MP/MPCM displayed good pharmacokinetics, which significantly prolonged the elimination phase half-life period (T_1/2β_), increased the maximum plasma concentration (C_max_) and reduced the clearance rate (Cl). This is because macrophage cell membrane coating could help nanocarriers to escape the immune system and prolong blood-circulation life time.

Next, the in vivo biodistribution experiment was performed, which was monitored with live fluorescence images of tumor-bearing nude mice. The results showed that after tail vein administration, the fluorescence intensity at the tumor sites gradually increased, reaching a peak 24 h later, and then gradually decreased (Fig. 4B). The fluorescence intensity of FA-MSNR/LMDI@MP/MPCM at the tumor site was stronger than that of the other three groups at all of the time points, and it lasted up to a certain extent until 72 h after administration, while the FA-MSN/LMDI group had the weakest and shortest duration of the four groups. Then, ex vivo organ fluorescence imaging was employed to examine the distribution of nanocarriers in various organs and tumors at 24 h postinjection. The results were consistent with the living images, in which the FA-MSNR/LMDI@MP/MPCM group showed the highest intensity in tumors, while the levels were low in other main organs, especially the liver and spleen (Fig. 4C, D).

Collectively, these findings demonstrated that FA-MSNR/LMDI@MP/MPCM could effectively promote targeted aggregation to tumors, increase tumor cell uptake, prolong the retention time of drugs in tumor tissue, and decrease the distribution of drugs in other organs.

### Photothermal and photodynamic effects and DOX release

**Fig. 5:**
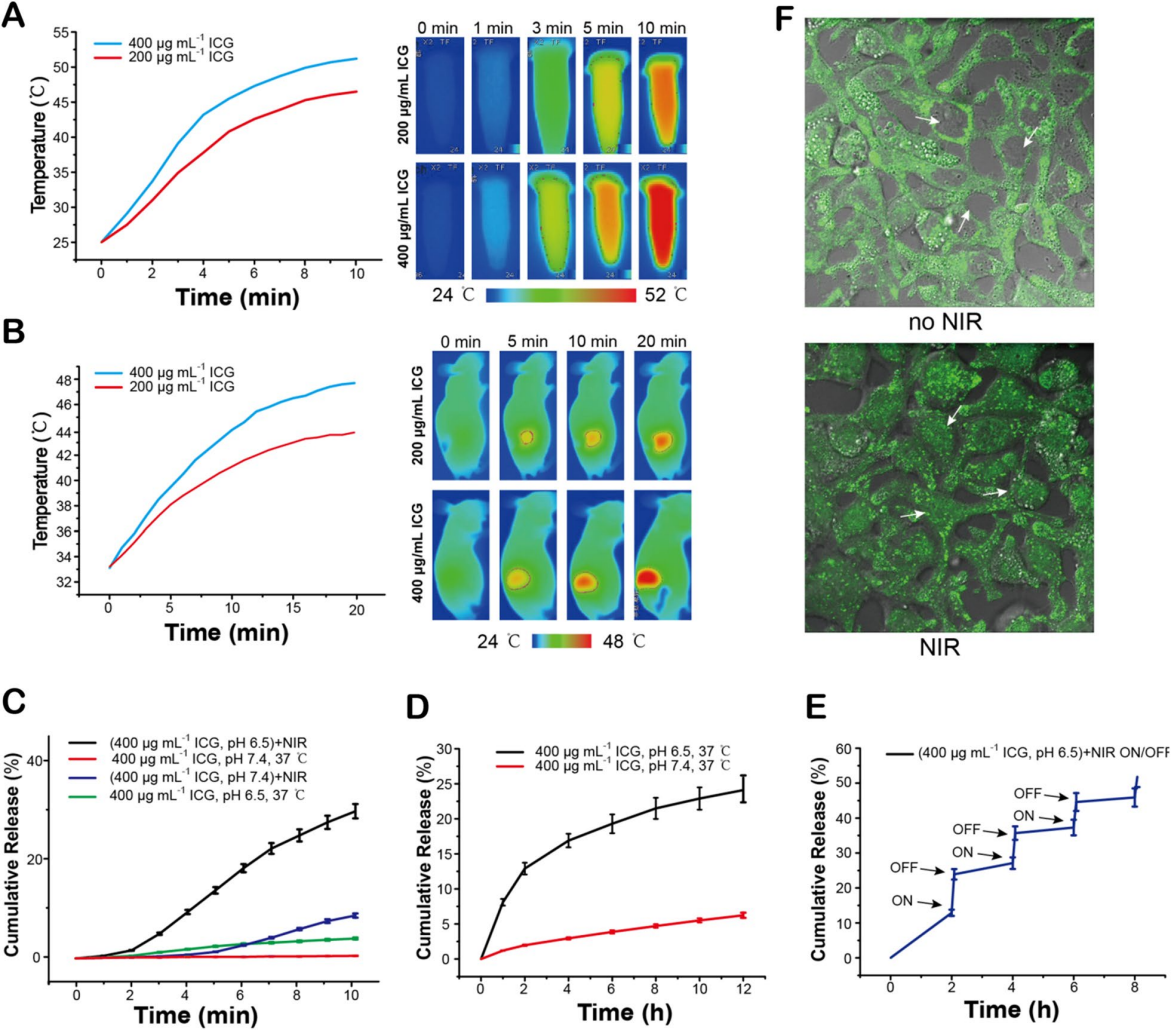
Photothermal effective and accumulative DOX release of FA-MSNR/LMDI@MP/MPCM. **A** Temperature change curves and infrared thermal images of FA-MSNR/LMDI@MP/MPCM with NIR irradiation. **B** Temperature change curves and infrared thermal images of tumor-bearing nude mice after intravenous injection of FA-MSNR/LMDI@MP/MPCM with NIR irradiation. **C** Accumulative Dox release curves of FA-MSNR/LMDI@MP/MPCM containing 400 μg mL^−1^ ICG at 37 °C, pH 7.4 or pH 6.5, with or without NIR irradiation for 10 min. **D** Accumulative Dox release curves of FA-MSNR/LMDI@MP/MPCM containing 400 μg mL^−1^ ICG within 12 h at 37 °C, pH 7.4 or pH 6.5. **E** Cumulative DOX release from FA-MSNR/LMDI@MP/MPCM in response to NIR irradiation on/off cycle. **F** LSCM images of 4T1 cells incubated with FA-MSNR/LMDI@MP/MPCM before and after NIR irradiation. (The arrow showed the nucleus) Green: DOX

Given the properties of ICG, the photothermal effect of FA-MSNR/LMDI@MP/MPCM was examined in vitro. A test tube containing FA-MSNR/LMDI@MP/MPCM (400 μg mL^−1^ ICG) was exposed to NIR, and its temperature rose from 25.0 to 45.5 °C after 5 min. After 10 min, a temperature of 51.2 °C was achieved. As the concentration of ICG decreased, its photothermal effect also declined (Fig. 5A). We then studied its photothermal effect in vivo. tumor-bearing nude mice were subjected to NIR for 20 min 24 h post FA-MSNR/LMDI@MP/MPCM injection into the tail vein. Prior to NIR irradiation, the tumor surface temperature was approximately 33 °C. During the course of irradiation, the temperature reached 47.7 °C for 20 min (Fig. 5B).

**Fig. 6:**
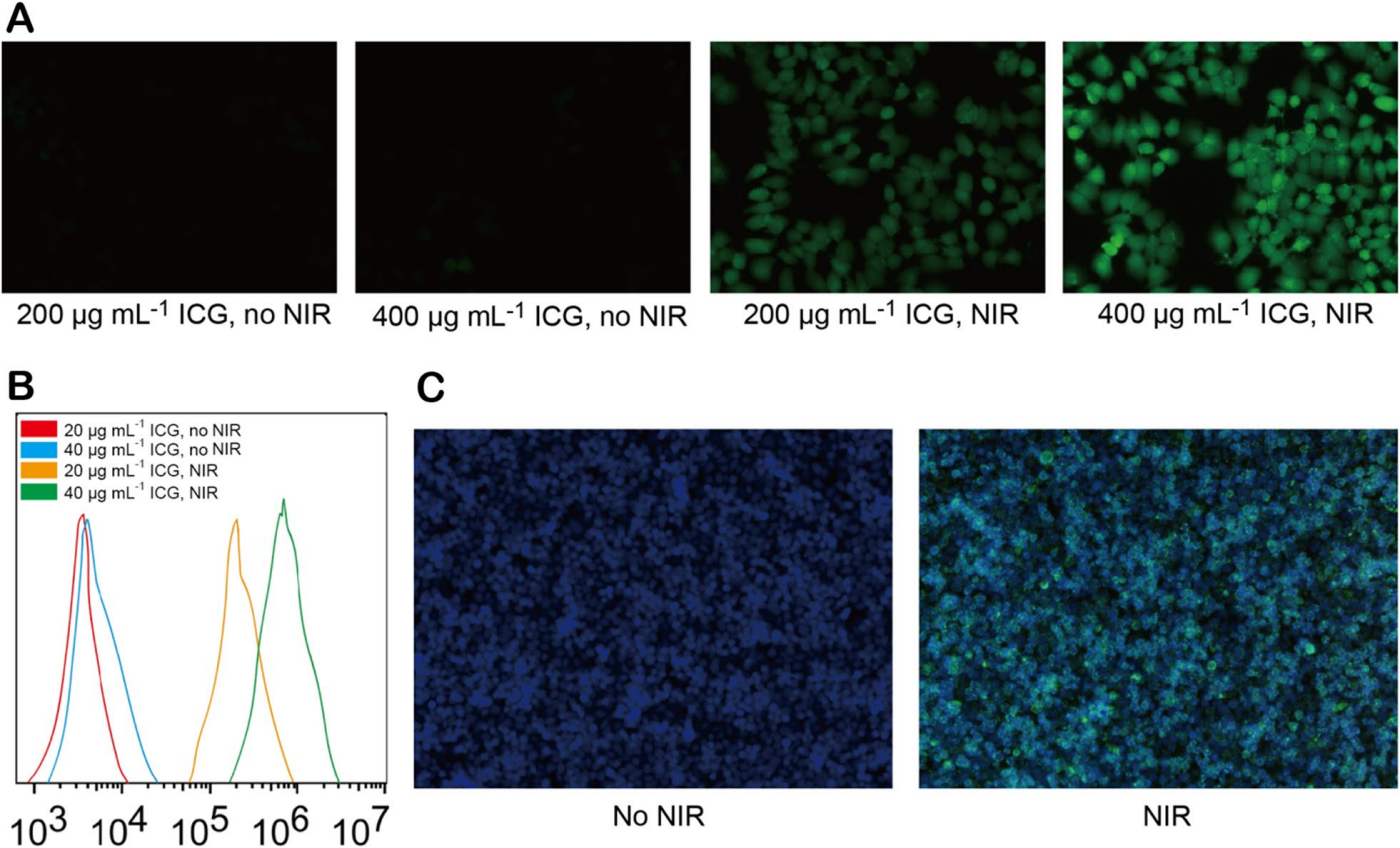
Photodynamic effective of FA-MSNR/LMDI@MP/MPCM. **A** LSCM images of 4T1 cells incubated with FA-MSNR/LMDI@MP/MPCM for DCFH-DA detection before and after NIR irradiation. Green: DCFH-DA. **B** Flow cytometry measurements of 4T1 cells incubated with FA-MSNR/LMDI@MP/MPCM for DCFH-DA signals before and after NIR irradiation. **C** LSCM images of the tumor sections of tumor-bearing nude mice injected after intravenous injection of FA-MSNR/LMDI@MP/MPCM for DCFH-DA detection with or without NIR irradiation. Blue: DAPI; green: DCFH-DA

Intracellular ROS can oxidize 2,7-dichlorofluorescein diacetate (DCFH-DA) into a highly fluorescent DCF molecule. Since the fluorescence intensity of DCF is directly proportional to the amount of intracellular ROS, the DCFH-DA probe was employed to evaluate intracellular ROS production. The green fluorescence of DCFH-DA was observed and detected through laser scanning confocal microscopy (LSCM) and flow cytometry. As shown in Fig. 6A, B, there were no obvious fluorescence signals in the cells incubated with FA-MSNR/LMDI@MP/MPCM without NIR irradiation, but the green fluorescence intensities were strong following NIR exposure. Similarly, ex vivo analysis of dissected tumor tissue also exhibited strong green fluorescence following irradiation (Fig. 6C). These results suggested that FA-MSNR/LMDI@MP/MPCM had good photodynamic effects in vitro and in vivo.

In the physiology-mimicking milieu (pH 7.4, 37 °C), the DOX release rate of FA-MSNR/LMDI@MP/MPCM was low. The release rate reached 6.2% after 12 h, while at pH 6.5 (37 °C), the DOX release rate was 24.1% after 12 h (Fig. 5C). This result revealed that MPEG-PAE and macrophage cell membrane coatings could also prevent drug release in addition to LM. Furthermore, when NIR irradiation was performed at pH 6.5, DOX release significantly accelerated and reached 29.2% 10 min later (Fig. 5D). In contrast, when NIR irradiation was performed at pH 7.4, the DOX release rate was significantly lower and only reached 8.5% after 10 min. This difference was attributed to the LM phase transition temperature being reached when ICG was heated via NIR irradiation, which subsequently led to pore opening and the rapid release of the loaded DOX. Additionally, in a weakly acidic environment (pH 6.5), MPEG-PAE swelling resulted in detachment of the macrophage cell membrane, which further accelerated DOX release. Compared with the Dox release curves of FA-MSNR/LMDI and FA-MSNR/LMDI@MPCM (Additional file 1: Figure S8), FA-MSNR/LMDI@MP/MPCM exhibted excellent thermosensitive and pH-sensitive release performances.

Subsequently, the release behaviours of FA-MSNR/LMDI@MP/MPCM with alternating NIR irradiation on/off cycles were determined. Figure 5E showed that pulsatile release could be detected, indicating powerful NIR-triggered prompt release.

Before NIR irradiation, the green fluorescence of DOX was observed in the cytoplasm (Fig. 5F) because FA-MSNR/LMDI@MP/MPCM also predominantly accumulated in the cytoplasm. When NIR irradiation was not carried out, the loaded DOX was not released as free DOX and could not enter the nucleus to exert its cytotoxic effect. However, following irradiation, DOX was released into the nucleus, where it was able to initiate cellular apoptosis. These results indicated that NIR irradiation could synchronize photothermal effects and DOX release to achieve the synergistic effect of photothermal chemotherapy.

### Biocompatibility and cytotoxicity of MSNRs

Cytotoxicity and biocompatibility are issues that should be considered when designing nanocarriers. When examining the hemolysis percentages in MSNR samples at concentrations ranging from 100 μg mL^−1^ to 1000 μg mL^−1^, the percentages were lower than the international standard of 5% (Additional file 1: Figure S9A), indicating that MSNRs had excellent hemo-compatibility.

Next, the stability of MSNRs in human blood serum was tested. Additional file 1: Figure S9B showed that the size distribution of nanoparticles changed littlen within 14 days, indicating their good stability in human blood serum.

**Fig. 7:**
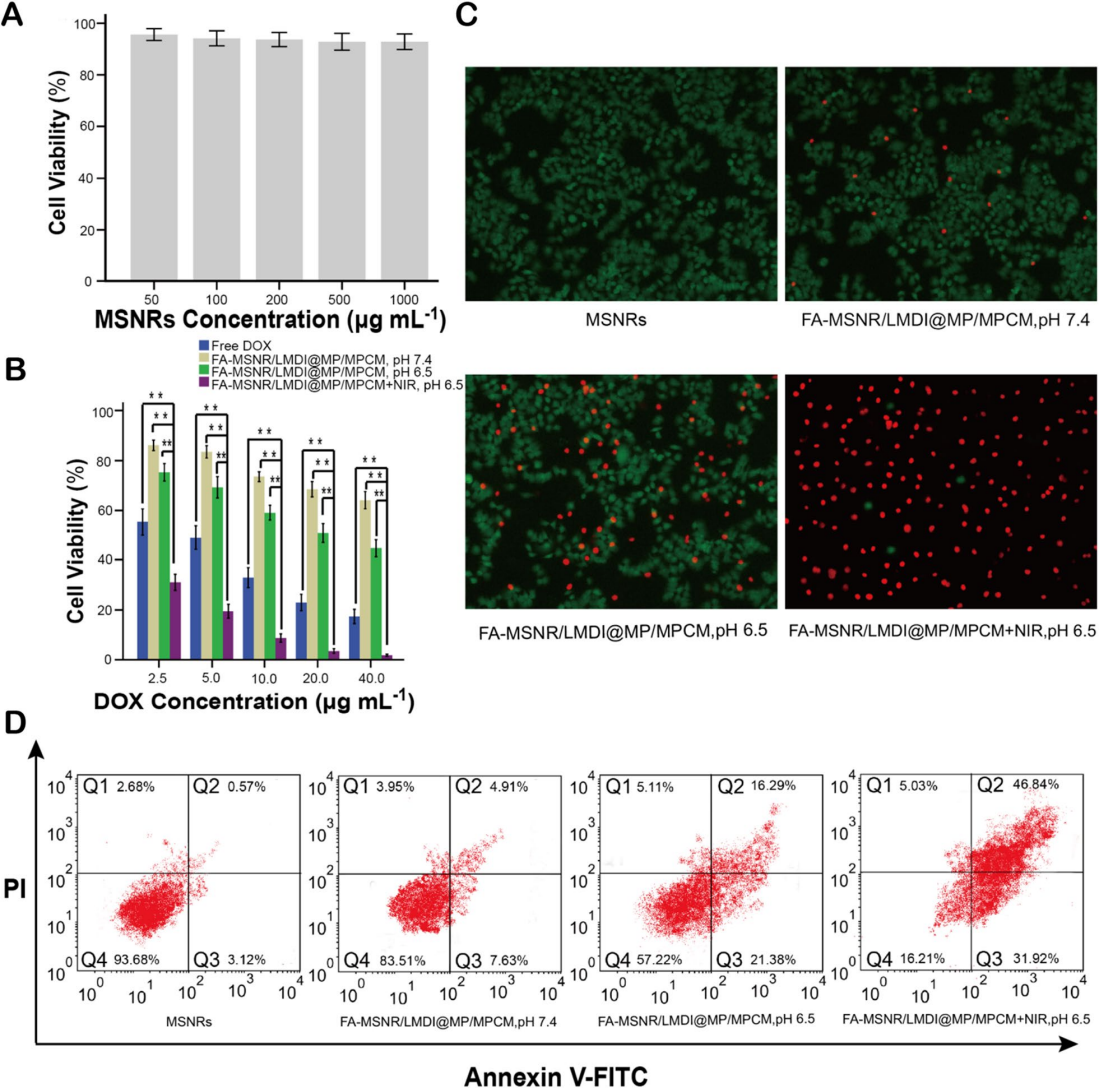
In vitro cytotoxicity of FA-MSNR/LMDI@MP/MPCM. **A** Cytotoxicity of various concentrations of MSNRs in 4T1 cells. **B** Cytotoxicity of FA-MSNR/LMDI@MP/MPCM with various DOX concentrations on 4T1 cells at pH 7.4 or pH 6.5, with or without NIR irradiation. **C** Fluorescence images of live and dead 4T1 cells treated with various methods. Green: live cells; red: dead cells. **D** Flow cytometric analysis of 4T1 cell apoptosis by staining with Annexin-V-FITC/PI. *P < 0.05, **P < 0.01 was analyzed by one way analysis of variance (ANOVA)

We used MTT to detect the cytotoxicity of MSNRs. After incubation with MSNRs for 48 h, the Mice breast cancer cells (4T1), human hepatocytes (L-02) and human embryonic kidney cells (293T) viabilities were more than 90% (Fig. 7A, Additional file 1: Figure S9C, D), exhibiting no significant cytotoxicity.

Then, blood samples of nude mice injected with MSNRs were collected to detect liver and kidney function indices. We found that even when the doses of MSNRs reached 10.0 mg kg^−1^, there were no significant differences between the experimental group and the control group (Additional file 1: Table S2). This demonstrated that MSNRs did not damage liver or kidney function. Subsequently, H&E staining of the main organs from nude mice injected with MSNRs displayed no obvious lesion areas (Additional file 1: Figure S10A). MSNRs induced no notable toxicity to the heart, liver, spleen, lung or kidney. These results indicated that the MSNRs had good biocompatibility and low cytotoxicity.

### In vitro and in vivo antitumor efficacy studies

Then, the killing effect of FA-MSNR/LMDI@MP/MPCM on tumor cells was evaluated in vitro. Without NIR irradiation, the DOX release rate of FA-MSNR/LMDI@MP/MPCM was very low, and the macrophage cell membrane coating hindered tumor cell phagocytosis. Therefore, in treated 4T1 cells (pH 7.4), and FA-MSNR/LMDI@MP/MPCM had the lowest growth inhibition rate, while the free DOX treatment had the highest (Fig. 7B). This finding indicated that the cytotoxicity of FA-MSNR/LMDI@MP/MPCM was relatively low under physiological conditions. Under pH 6.5 and NIR irradiation, the cytotoxicity of FA-MSNR/LMDI@MP/MPCM was markedly increased, and the cell viability was significantly decreased; it was lower than that of tumor cells incubated with free DOX at the same concentration (Fig. 7B).

The survival state of tumor cells can be visualized directly by AM-PI live (green) and dead (red) staining (Fig. 7C). The FA-MSNR/LMDI@MP/MPCM + NIR group (pH 6.5) displayed the most red-stained dead cells, followed by the FA-MSNR/LMDI@MP/MPCM group (pH 6.5) and FA-MSNR/LMDI@MP/MPCM group (pH 7.4), with the MSNR-treated group being predominantly green-stained living cells. We analysed apoptosis quantitatively through flow cytometry using Annexin-V-FITC/PI staining. Figure 7D shows that the percentages of early and late apoptotic cells (Q2 + Q3): from high to low, they were the FA-MSNR/LMDI@MP/MPCM + NIR group (pH 6.5) (78.76%), FA-MSNR/LMDI@MP/MPCM group (pH 6.5) (37.67%), FA-MSNR/LMDI@MP/MPCM group (pH 7.4) (12.54%) and MSNRs (3.69%). These findings were the results of a weakly acidic environment, in which the nanocarriers separated from the macrophage cell membrane coating to promote the uptake of tumor cells and then synergistically killed the tumor cells through the combined PTT and PDT effects of ICG and the released DOX.

**Fig. 8:**
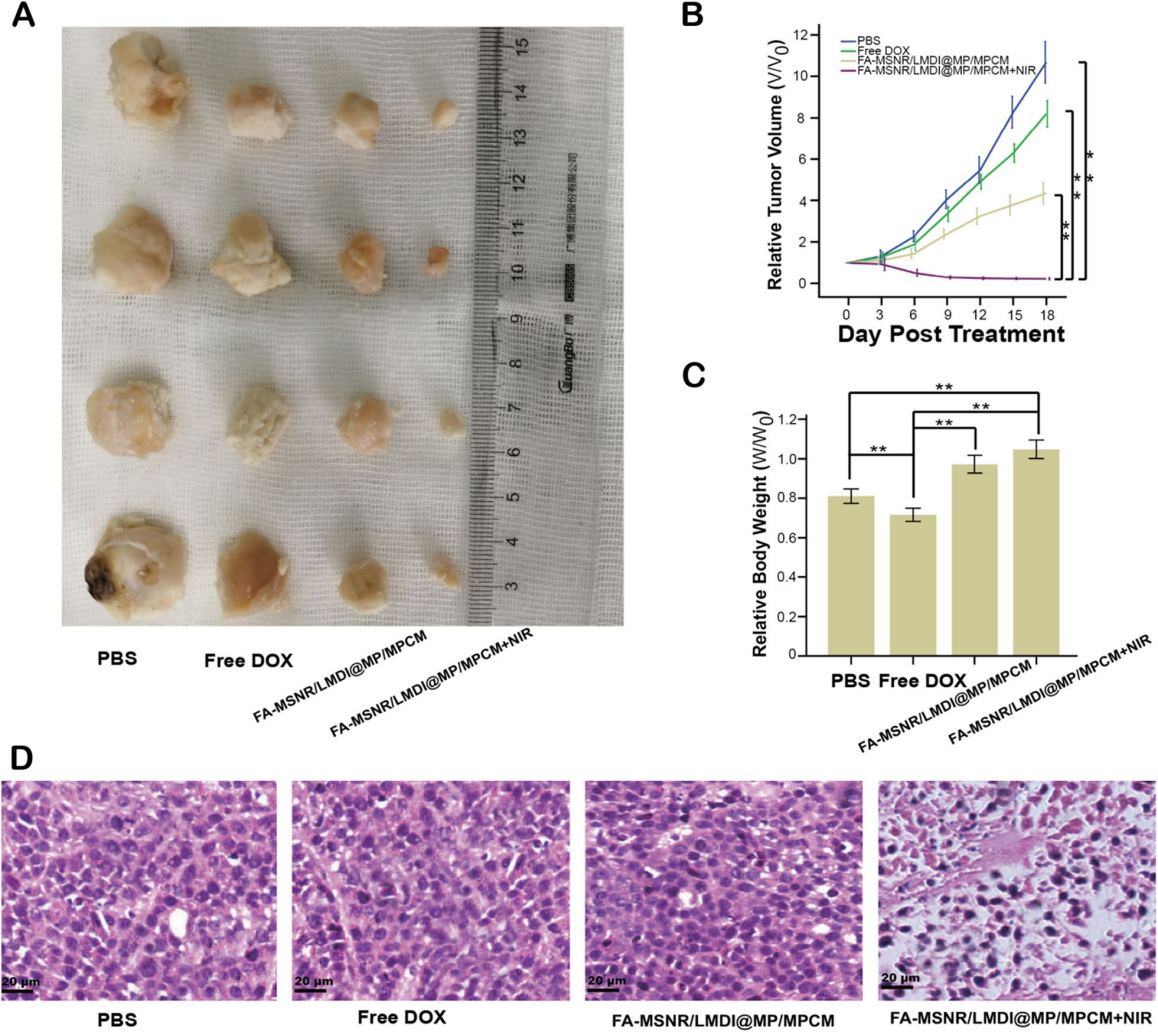
In vivo antitumor effect of FA-MSNR/LMDI@MP/MPCM. **A** Photos of the tumor tissues of various groups after treatments (day 18). **B** tumor growth curves of various groups at the end of treatment. **C** Relative body weights of the mice after various treatments. **D** H&E staining of tumor slices of the mice after various treatments. **P < 0.01 was analyzed by one way analysis of variance (ANOVA)

To evaluate the antitumor effect of FA-MSNR/LMDI@MP/MPCM in vivo, the tumor volume was measured. We divided tumor-bearing nude mice into 4 groups based on various experimental methods: (I) PBS; (II) free DOX; (III) FA-MSNR/LMDI@MP/MPCM and (IV) FA-MSNR/LMDI@MP/MPCM + NIR. At the end of treatment (Day 18), Group IV exhibited the highest relative tumor volume reduction, of approximately 85%. The tumors in the remaining three groups grew faster, and the tumors in Groups I and II increased to more than sevenfold that of the original tumor volume, while the tumors in Group III increased 5.5-fold. In contrast to the in vitro results, the cytotoxicity of FA-MSNR/LMDI@MP/MPCM was not as high as that of free DOX. Compared with free DOX, the tumor inhibitory rate of FA-MSNR/LMDI@MP/MPCM in vivo was higher (Fig. 8A, B). This result might have been due to the fast clearance, short retention time, and low tumor uptake of free DOX in vivo. Furthermore, previous experimental results showed that FA-MSNR/LMDI@MP/MPCM could increase tumor cell uptake and that DOX was slowly released at the tumor site; hence, FA-MSNR/LMDI@MP/MPCM had a better antitumor effect in vivo than free DOX. After treatment, H&E staining of tumor slices showed that the majority of tumor cells were seriously damaged (Fig. 8D), and there was a large area of coagulated necrosis in the tumor tissues of Group IV. Together, these results demonstrated that FA-MSNR/LMDI@MP/MPCM with NIR could obtain effective tumor suppression and destruction via synergistic treatment in vivo. In addition, as seen with percent survival curves (Additional file 1: Figure S11), the survival time of Group IV was significantly prolonged. Above results displayed that tumor-bearing nude mice treated with FA-MSNR/LMDI@MP/MPCM + NIR validated the commendable antitumor effect.

Moreover, the body weights of the nude mice in each group were found to be significantly different after treatment. Compared with Group I (PBS), the body weight of Group II (free DOX) decreased, but the body weights of Groups III and IV did not significantly decrease; the mice in Group IV even showed a slight increase (Fig. 8C). Moreover, the heart, liver, spleen, lung, and kidney of group IV mice had no obvious lesions (Additional file 1: Figure S10B), and there was no significant damage to liver or kidney function (Additional file 1: Table S2). These results indicated that FA-MSNR/LMDI@MP/MPCM had good biocompatibility and could effectively reduce DOX-related side effects.

## Conclusions

In summary, we designed a multilevel intelligent responsive nanodrug delivery system with a macrophage cell membrane coating to evade the immune system and facilitate tumor targeting and a rod-shaped MSNR to optimize deep tumor penetration. The pH-sensitive cationic polymer proton sponge effect was also employed to induce macrophage cell membrane detachment and thus increase tumor phagocytosis. This drug delivery system was based tissue-specific multilevel targeted accumulation, precisely controlled drug release, and the synergistic effects of PTT, PDT and chemotherapy to effectively kill tumor cells and reduce the toxic side effects of DOX. These experimental results showed that FA-MSNR/LMDI@MP/MPCM, as a new type of nanocarrier, has great potential for use in tumor therapy and may provide a new foundation for tumor targeted therapy.

## Supplementary Information

**Additional file 1: ** Matierals, Parts of experiment section.** Figure S1.** TEM images of macrophage cell membrane and various nanocarriers. (A) Macrophage cell membrane. (B) Spherical MSNs. (C) FA-MSNR/LMDI@MPCM. (D) FA-MSN/LMDI. **Figure S2.** TEM images of various nanocarriers. (A) MSNRs. (B) FA-MSNR/LMDI. (C) FA-MSNR/LMDI@MPCM. (D) FA-MSNR/LMDI@MP/MPCM. (E) Spherical MSNs. (F) FA-MSN/LMDI. **Figure S3.** (A) Chemical structure of MPEG-PAE. (B) ^1^HNMR spectrum of MPEG-PAE. (C) GPC of MPEG-PAE. **Figure S4.** Changes in particle sizes of MPEG-PAE by DLS at various pH values. (A) pH 7.4. (B) pH 7.2. (C) pH 7.0. (D) pH 6.8. **Figure S5.** Flow cytometry measurements of uptake of the four types of nanocarriers by 4T1 cells and macrophages. **Figure S6.** Tumor sections of tumor-bearing nude mice 2, 4 and 24 h after injection with the four types of nanocarriers. Blue: DAPI; green: CD34; red: nanocarriers. **Figure S7.** Concentration of DOX in plasma at different time after intravenous injection of free DOX, FA-MSNR/LMDI and FA-MSNR/LMDI@MP/MPCM. **Figure S8.** Accumulative Dox release curves of FA-MSNR/LMDI (A) and FA-MSNR/LMDI@MPCM (B) containing 400 μg mL^−1^ ICG at 37 °C, pH 7.4 or pH 6.5, with or without NIR irradiation for 10 min. **Figure S9.** (A) In vitro hematological analysis of MSNRs with various concentrations. (B) Stability analysis of MSNRs in human blood serum. (C) Cytotoxicity of MSNRs with various concentrations on L-02 cells. (D) Cytotoxicity of MSNRs with various concentrations on 293T cells. **Figure S10.** Histology stainings of main organs from tumor-bearing nude mice. (A) H&E stainings of the sections of heart, liver, spleen, lung, and kidney from tumor-bearing nude mice after intravenous injection of MSNRs. (B) H&E stainings of the sections of heart, liver, spleen, lung, and kidney from tumor-bearing nude mice after intravenous injection of FA-MSNR/LMDI@MP/MPCM with NIR irradiation. **Figure S11.** Percent survival for difffferent treatment groups during 50 days. **Table S1.** Pharmacokinetic parameters of DOX after intravenously administration of the four types of nanocarriers at the DOX dose of 1 mg kg^−1^ of mouse body weight. **Table S2.** Liver and kidney Function Test.

## Data Availability

All data generated or analyzed during this study are included in this published article.

## Abbreviations

MSNs: Mesoporous silica nanoparticles
MSNRs: Mesoporous silica nanorods
EPR: Enhanced permeation and retention
RES: Reticuloendothelial system
MPEG-PAE: Methoxy poly(ethylene glycol)-poly (β-amino ester)
ICG: Indocyanine green
ROS: Reactive oxygen species
PTT: Photothermal therapy
PDT: Photodynamic therapy
FA: Folic acid
DOX: Doxorubicin
LM: L-menthol
TEOS: Tetraethyl orthosilicate
TM: Transition temperature
