# Supplementary material for: An intelligent responsive macrophage cell membrane-camouflaged mesoporous silicon nanorod drug delivery system for precise targeted therapy of tumors
Source: J Nanobiotechnology. 2021 Oct 24;19:336. doi: 10.1186/s12951-021-01082-1 (PMC8543955; doi:10.1186/s12951-021-01082-1)
Supplement: Supplementary file 1 — Additional file 1: Matierals, Parts of experiment section. Figure S1. TEM images of macrophage cell membrane and various nanocarriers. (A) Macrophage cell membrane. (B) Spherical MSNs. (C) FA-MSNR/LMDI@MPCM. (D) FA-MSN/LMDI. Figure S2. TEM images of various nanocarriers. (A) MSNRs. (B) FA-MSNR/LMDI. (C) FA-MSNR/LMDI@MPCM. (D) FA-MSNR/LMDI@MP/MPCM. (E) Spherical MSNs. (F) FA-MSN/LMDI. Figure S3. (A) Chemical structure of MPEG-PAE. (B) 1HNMR spectrum of MPEG-PAE. (C) GPC of MPEG-PAE. Figure S4. Changes in particle sizes of MPEG-PAE by DLS at various pH values. (A) pH 7.4. (B) pH 7.2. (C) pH 7.0. (D) pH 6.8. Figure S5. Flow cytometry measurements of uptake of the four types of nanocarriers by 4T1 cells and macrophages. Figure S6. Tumor sections of tumor-bearing nude mice 2, 4 and 24 h after injection with the four types of nanocarriers. Blue: DAPI; green: CD34; red: nanocarriers. Figure S7. Concentration of DOX in plasma at different time after intravenous injection of free DOX, FA-MSNR/LMDI and FA-MSNR/LMDI@MP/MPCM. Figure S8. Accumulative Dox release curves of FA-MSNR/LMDI (A) and FA-MSNR/LMDI@MPCM (B) containing 400 μg mL−1 ICG at 37 °C, pH 7.4 or pH 6.5, with or without NIR irradiation for 10 min. Figure S9. (A) In vitro hematological analysis of MSNRs with various concentrations. (B) Stability analysis of MSNRs in human blood serum. (C) Cytotoxicity of MSNRs with various concentrations on L-02 cells. (D) Cytotoxicity of MSNRs with various concentrations on 293T cells. Figure S10. Histology stainings of main organs from tumor-bearing nude mice. (A) H&E stainings of the sections of heart, liver, spleen, lung, and kidney from tumor-bearing nude mice after intravenous injection of MSNRs. (B) H&E stainings of the sections of heart, liver, spleen, lung, and kidney from tumor-bearing nude mice after intravenous injection of FA-MSNR/LMDI@MP/MPCM with NIR irradiation. Figure S11. Percent survival for difffferent treatment groups during 50 days. Table S1. Pharmacokinetic pa [file 12951_2021_1082_MOESM1_ESM.docx]

**Supplementary Information**

# An intelligent responsive macrophage cell membrane-camouflaged mesoporous silicon nanorod drug delivery system for precise targeted therapy of tumors

Minghua Li^1^, Xiaolong Gao^2,3^, Chao Lin^4^, Aijun Shen^1^, Jing Luo^1^, Qiongqiong Ji^1^, Jiaqi Wu^1^, Peijun Wang^1,*^

^1^Department of Radiology, Tongji Hospital, School of Medicine, Tongji University, Shanghai 200065, P.R. China.

^2^Department of Radiology, Baoshan District, Luodian Hospital, Shanghai 201908, P.R. China.

^3^Department of Radiology, Luodian Hospital, Shanghai University, Shanghai 200444, P.R. China.

^4^Institute for Translational Medicine, Shanghai East Hospital, Institute for biomedical Engineering and Nanoscience, School of Medicine, Tongji University, Shanghai 200092, P.R. China

^*^ Correspondence: [wangpeijuntjyy@sina.com](mailto:wangpeijuntjyy@sina.com)

Department of Radiology, Tongji Hospital, School of Medicine, Tongji University, Shanghai 200065, P.R. China

**Matierals**

Cetyltrimethylammonium bromide (CTAB), tetraethyl orthosilicate (TEOS), aqueous ammonia (NH_3_^.^H_2_O), dimethylformamide (DMF), 3-aminopropyl triethoxysilane (APTES), 3-[4, 5-dimethylthiazolyl-2]-2, 5-diphenyltetrazolium bromide (MTT), CD34 monoclonal antibady and L-menthol (LM) were purchased from Sigma.

Methoxy poly(ethylene glycol) (mPEG) was purchased from Shanghai Aladdin Biochemical Technology Co., Ltd.1,6-Hexanediol diacrylate (HDD), 1,3-Bis(4-piperidyl) propane (TDP), 4-Methylpiperidine (MP), and thioridazine (Thz) were purchased from Alfa Aesar Chemistry Co. Ltd.

Indocyanine green (ICG) and doxorubicin (DOX) were purchased from Sinopharm Chemical Reagent Co., Ltd.

Annexin-V/PI Apoptosis Detection kit was purchased from ComWin Biotech Co., Ltd.

2,7′-Dichloroflfluorescein diacetate (DCFH-DA) was purchased from Tianjin Heowns Biochemical Technology Co., Ltd.

Dulbecco’s modifified Eagle’s medium (DMEM), fetal bovine serum (FBS), phosphate Buffered Saline (PBS), and trypsin were purchased from Gibco.

**Cells and animals**

Mice breast cancer cells (4T1), human hepatocytes (L-02) and human embryonic kidney cells (293T) were purchased from Shanghai cell bank of Chinese Academy of Sciences. Male BALB/c mice were purchased from Shanghai Jiesijie Experimental Animal Co. Ltd. (China)

**Methods**

**Synthesis of methoxy poly(ethylene glycol)-poly (β-amino ester) (MPEG-PAE)**

Synthesis of PBAE was synthesized via a Michael step polymerization. TDP (1.1 eq.), HDD (1.0 eq.), and MP (0.1 eq.) were co-dissolved in chloroform. The reactant was stirred at 60 ℃ for 48 h under nitrogen. After the reaction, chloroform was removed by reduced pressure. Then, the residual reaction residue was precipitated in cold diethyl ether for 12 h, and dried under vacuum for 48 h.Final light-yellow solids were obtained after filtration.

Synthesis of mPEG-PBAE was still synthesized via a Michael step polymerization. The mixture of mPEG (1 eq.) and PBAE (1 eq.) were stirred at 60 ℃ for 48 h under nitrogen. At the end of the reaction, the resulting solution was concentrated and transferred to a dialysis bag (Mw 5000) against an excess of distilled water for 48 h. Lastly, the solution was freeze-dried to obtain PEG-PBAE powder.

**Separation of mononuclear macrophage cell membrane**

First, mononuclear macrophage cells (cell number ≈ 1 × 10^7^ ) were isolated from culture dish with 0.25% Trypsin-EDTA and washed with PBS three times, and dispersed in PBS. Next, a hypotonic lysing buffer consisting of 1 mmol L^-1^ NaHCO_3_, 0.2 mmol L^-1^ EDTA and 1 mmol L^-1^ PMSF, which were added in cells suspension solution and kept in 4 °C over night. Cell suspended solution were repeatedly grinding with a tight-fitting for 20 times before 3200 g, 5 min, 4 ℃ centrifuge. The pellet was removed, then the supernatant was centrifuged for 100000 g 30 min in 4 ℃, and supernatant was discarded. The eventual pellet was macrophage cell membrane (MPCM) ghosts.

**Characterization and properties of nanocarriers**

Various samples were monitored by UV−vis spectroscopy (UV-Vis-NIR, Cary 5000, Agilent, USA). Morphology was visualized by transmission electron microscopy (TEM) (JEM-2010F, JEOL, Japan). The average particle size, and Zeta potential measurements were all obtained using a Malvern Mastersizer (Nano ZS, Malvern Instruments, U.K.) at different pH. The Brunauer−Emmett−Teller (BET) surface area and pore size of MSNRs and FA-MSNR/LMDI were measured by a BET Tester (Gemini VII2390, Micromeritics, USA) using nitrogen adsorption−desorption curve and Barrett−Joyner−Halenda (BJH) methods, respectively.

UV/Vis was used to measure the absorbance of the FA-MSNR/LMDI solution and the standard curve was plotted to calculate the drug loading content (DLC) of the nanocarrier. Drug Loading Content (DLC) (%) = Total DOX (or ICG) Dosage - Unencapsulated DOX (or ICG) Dosage / Total Mass of Nanocarrier × 100%

**^1^HNMR Characterization of MPEG-PAE**

Before ^1^HNMR characterization, the MPEG-PAE sample was dried overnight in a vacuum oven. Next, 5 mg of the sample was weighed out, and 0.5 mL of deuterated DMSO or deuterated chloroform was added to the sample with a pipette. The mixture was ultrasonicated for half an hour. After the sample had completely dissolved, its ^1^HNMR was measured with an Avance 500 MHz NMR spectrometer (Bruker BioSpin, Switzerland).

**GPC of MPEG-PAE**

GPC was performed using KF-803L and KF-802.5 (Shodex, Japan) columns in series with tetrahydrofuran (THF) as the eluent at a flow rate of 1 ml/min. The data was analyzed by means of an RI detector (RI-101, Shodex, Japan), wherein PEG standards were used to calculate the polymer molecular weight.

**Cells culture and tumor modeling**

Mouse breast cancer 4T1 cells cells were cultured in DMEM containing 10% FBS and 1% of the double-antibiotic (penicillin and streptomycin) in an incubator supplied with 5% CO_2_ at 37 °C. Cells were passaged every 3–4 d. The cells at the logarithmic growth phase were suspended in PBS cells at a density of 1×10^7^ cells mL^-1^. The 4-week-old nude mice were anesthetized with an intraperitoneal injection of 10% chloral hydrate, followed by slow injection of 0.3 mL of the 4T1 cells suspension into the subcutaneous right hind legs. The mice were ready for experimentation when the tumors grew to a diameter of approximately 1 cm. The institutional Animal Care and Use Committees on Animal Care of Tongji Hospital of Tongji University approved our animal protocols prior to start of the experiments.

**Intracellular uptake of nanocarriers**

Upon achieving 80% confluency, the four types of nanocarriers were incubated with 4T1 cells and macrophages for 4 h. The culture medium with 4T1 cells was pH 7.4 and 6.5, respectively. After incubation for 4 h, a portion of the cells was measured using flow cytometry (C6, BD, USA) based on ICG fluorescence; the rest of the cells were stained with DAPI, and laser scanning confocal microscopy (LSCM) (TCS SP5, Leica, Germany) was then used to observe the intracellular distribution of nanocarriers. 4T1 cells incubated with FA-MSNR/LMDI@MP/MPCM (pH 7.4) for 1 h, 2 h and 4 h, they were prepared into ultrathin sections to be seen by TEM.

**Photothermal effect of FA-MSNR/LMDI@MP/MPCM**

FA-MSNR/LMDI@MP/MPCM (200, 400 μg mL^-1^ ICG) was irradiated with NIR (808 nm, 1.5 W cm^-2^) for 10 min. We recorded the thermal images with an infrared thermal camera (RC05, Rinch, Hongkong China) and measured the temperature. We injected FA-MSNR/LMDI@MP/MPCM (200, 400 μg mL^-1^ ICG) into tail veins of tumor-bearing nude mice. After 24 h, they were irradiated with NIR (808 nm, 1.5 W cm^-2^) for 20 min. The thermal effects were observed and measured using an infrared thermal camera.

**Photodynamic effect of FA-MSNR/LMDI@MP/MPCM**

4T1 cells were incubated with FA-MSNR/LMDI@MP/MPCM (200, 400 μg mL^-1^ ICG) for 4 h. After washing with PBS, DCFH-DA solution was added and the solution was incubated with the 4T1 cells for 15 min. When irradiated with NIR (808 nm, 1.5 W cm^-2^), the cells were observed using fluorescence microscope or collected and analyzed using flow cytometry.

FA-MSNR/LMDI@MP/MPCM (400 μg mL^-1^ ICG) was injected into tumor-bearing nude mice. After 24 h, A DCFH-DA solution was directly injected into the tumor tissiue. Afterward, NIR irradiation (808 nm, 1.5 W cm^-2^) was performed on mice, mice without NIR as a control. Subsequently, Tumor slices from two groups were observed by fluorescence microscope.

**In vivo pharmacokinetic assay.**

12 nude mice were randomly divided into 3 groups, and they were fasted overnight before the experiment. The solution of FA-MSNR/LMDI@MP/MPCM, FA-MSNR/LMDI and free DOX was injected through caudal vein (DOX dose of 1,000 μg kg^-1^ of mouse body weight ) . The blood samples were obtained at different time points . DOX was extracted by dissolving blood samples in HCl (0.75 M)/isopropanol at -20 °C overnight and the amount of DOX in the plasma was evaluated by fluorescence absorption. The plasma clearance (Cl) and the area under the blood concentration curve (AUC), which was the main pharmacokinetic parameters, were calculated using WinNonlin 3.3 software.

**DOX release rate of FA-MSNR/LMDI@MP/MPCM**

FA-MSNR/LMDI@MP/MPCM, FA-MSNR/LMDI and FA-MSNR/LMDI@MPCM (400 μg/mL ICG) solution was irradiated with or without NIR (808 nm, 1.5 1.5 W cm^-2^) at different pH (6.5, 7.4) for 10min. To study the release profile of DOX from FA-MSNR/LMDI@MP/MPCM in response to NIR irradiation on/off cycle, FA-MSNR/LMDI@MP/MPCM, FA-MSNR/LMDI and FA-MSNR/LMDI@MPCM (400 μg mL^-1^ ICG) solution was irradiated by NIR (808 nm, 1.5 1.5 W cm^-2^) at pH 6.5 for 10 min after 2, 4, 6 h. We used OPDA for measuring absorbance at time intervals that were set. Next we calulated the rate of release for DOX.

We studied the photothermal effect on the intracellular DOX distribution. After 4T1 cells were incubated with FA-MSNR/LMDI@MP/MPCM for 2 h, the cells were subjected to NIR for 2 min. LSCM was used to observe the intracellular distribution of DOX and its relationship with the nucleus based on DOX fluorescence.

**In vitro hematological analysis**

Blood was obtained from healthy New Zealand rabbits and anticoagulated with potassium oxalate. MSNRs detected were divided into four concentration groups, ie, 100, 200, 500, and 1000 μg mL^-1^. 0.9% saline and distilled water were used as negative and positive controls, respectively. Then, 0.2 mL of diluted anticoagulated blood was added to each tube preheated. After incubation for 60 min, the tubes were centrifugated for 5 min. Next, the supernatant fluid was assembled, and OD values were measured at 545 nm by UV-vis spectrophotometry. The hemolysisrate (HR) was calculated as follows: HR(%) = (OD of the experimental group-OD of negative control group)/(OD of the positive control group-OD of negative control group) × 100%.

**Stability analysis of MSNRs**

The in vitro stability evaluation was carried out based on the size of MSNRs in human blood serum. The human blood serum was separated by centrifugation (1500 rpm min^-1^, 10 min) to remove the red blood cells. First, MSNRs must be filtrated by 0.22 µm sterile membranes to move the bacterium and the disinfected size vessels were used to disperse the nanosystem by the human blood serum. Then, the size of MSNRs in different time was detected by DSL.

**Liver and kidney function of nude mice**

Blood samples were collected from superior orbital vein of each mouse after treatment. The blood samples were placed in sterile at 4 ℃ for 2 h, centrifuged at 3000 rpm for 20 min. The main biochemical indexes of liver and kidney function were measured as follows: aspartate aminotransferase (AST), alanine aminotransferase (ALT), alkaline phosphatase (ALP) Serum urea nitrogen (BUN) and creatinine clearance rate (CCR).

**Histopathological examination of main organs and tumors in nude mice**

After treatment, the mice in each group were sacrificed and the organs were separated from the bodies to measure the H&E staining. The heart, liver, spleen, lung, kidney and tumors were fixed in formalin and processed in paraffin. Then, the tissues were sliced at 4 μm thickness for H&E staining to monitor changes of each organ after treatment.


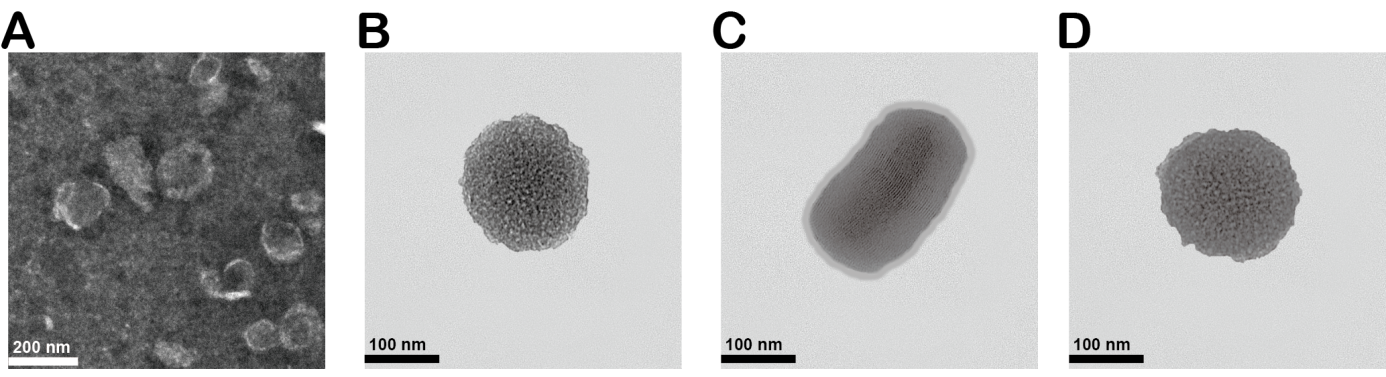


**Fig. S1** TEM images of macrophage cell membrane and various nanocarriers. **A** Macrophage cell membrane. **B** Spherical MSNs. **C** FA-MSNR/LMDI@MPCM. **D** FA-MSN/LMDI.


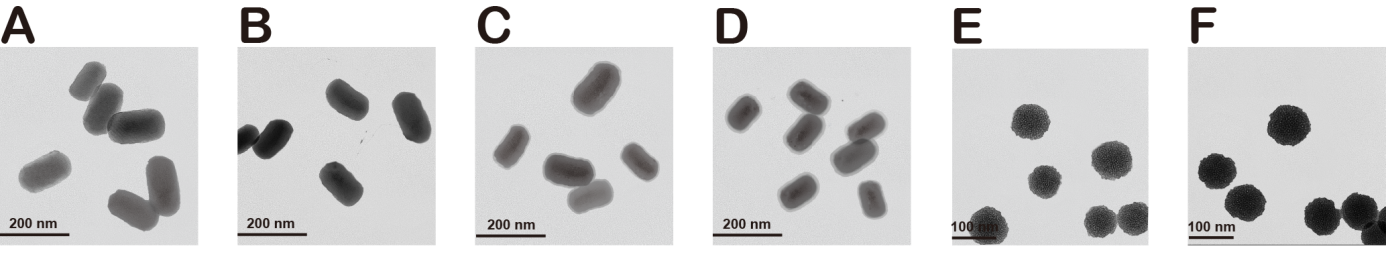


**Fig. S2** TEM images of various nanocarriers. **A** MSNRs. **B** FA-MSNR/LMDI. **C** FA-MSNR/LMDI@MPCM. **D** FA-MSNR/LMDI@MP/MPCM. **E** Spherical MSNs. **F** FA-MSN/LMDI.


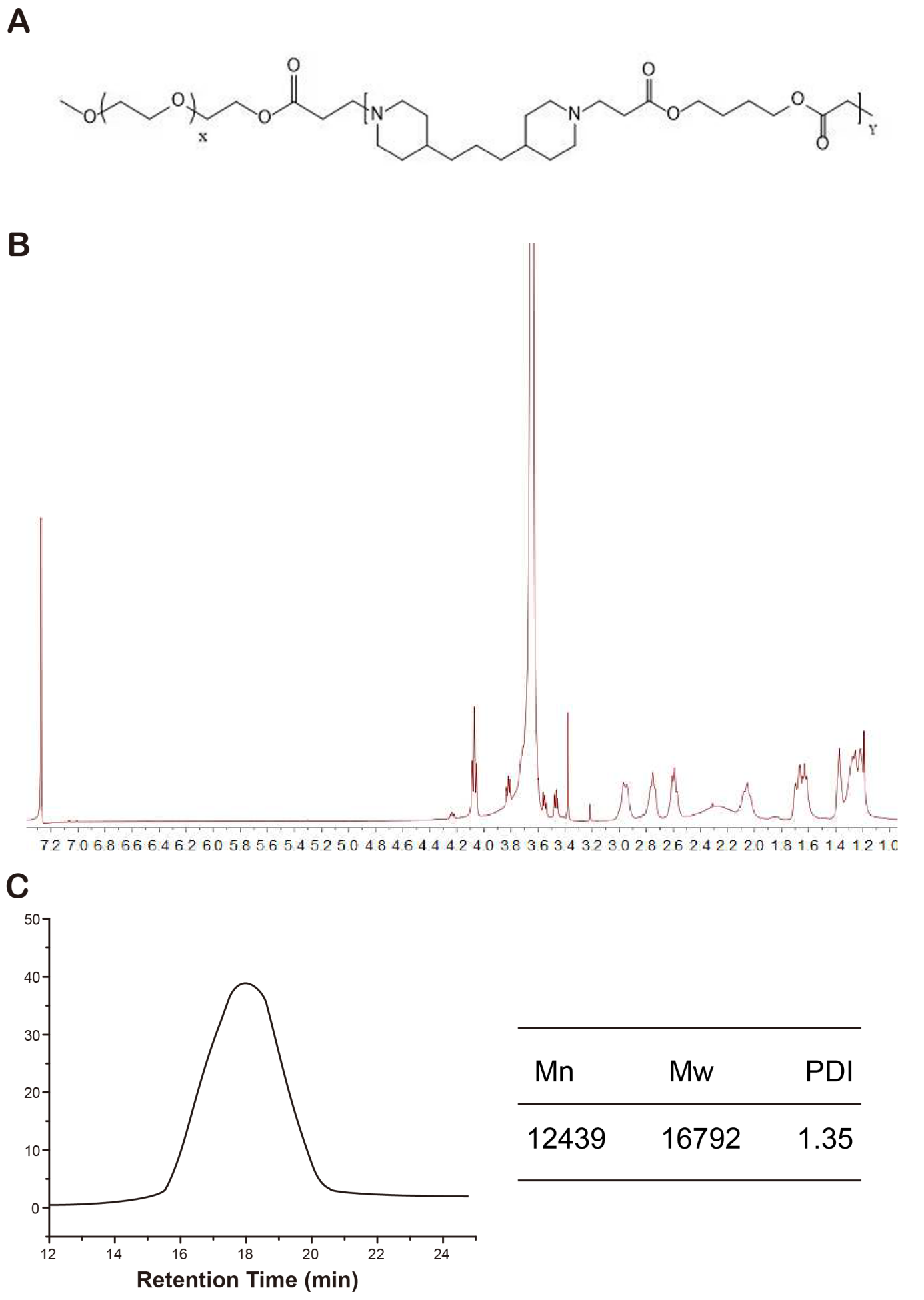


**Fig. S3 A** Chemical structure of MPEG-PAE. **B** ^1^HNMR spectrum of MPEG-PAE. **C** GPC of MPEG-PAE.


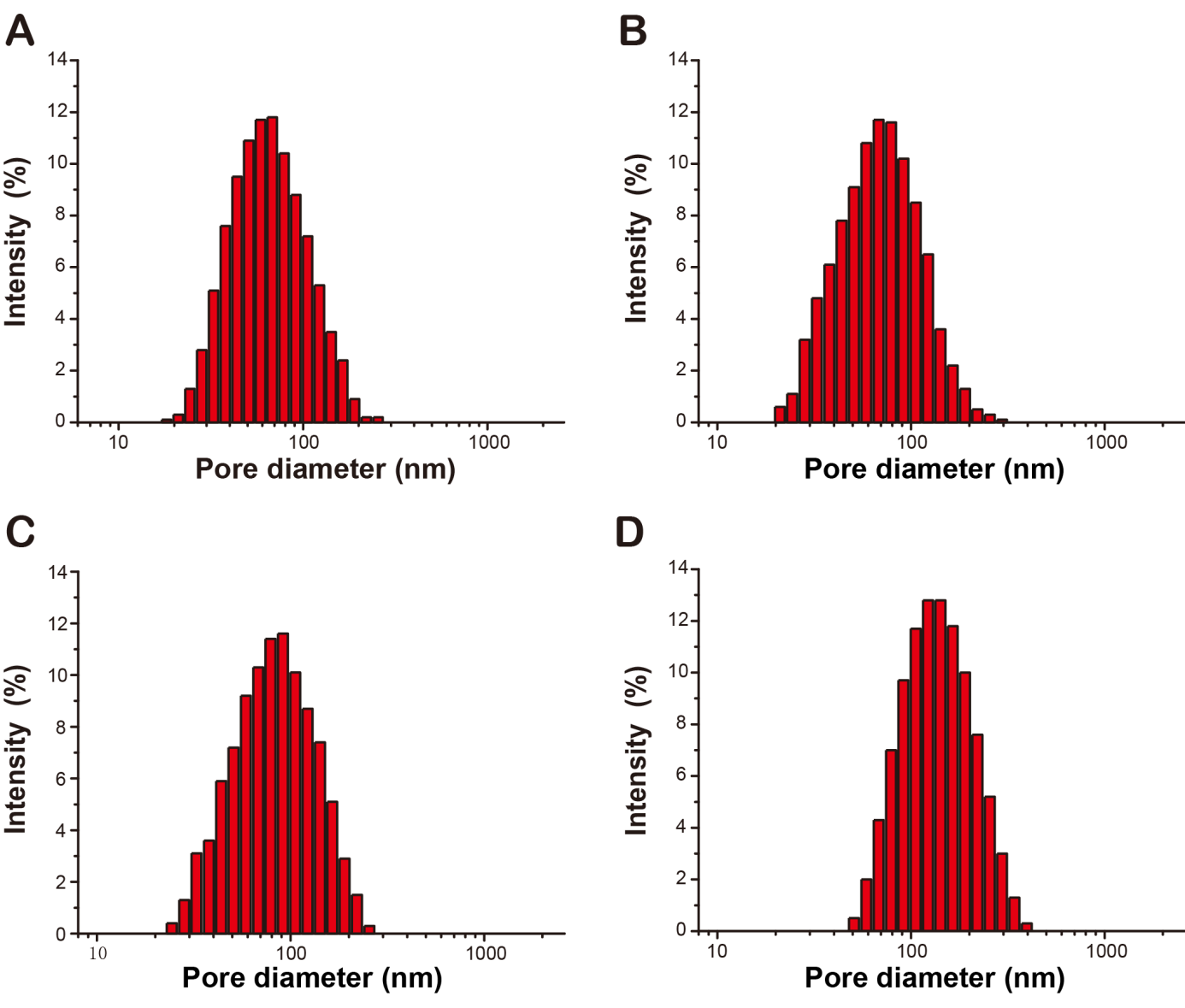


**Fig. S4** Changes in particle sizes of MPEG-PAE by DLS at various pH values. **A** pH 7.4. **B** pH 7.2. **C** pH 7.0. **D** pH 6.8.


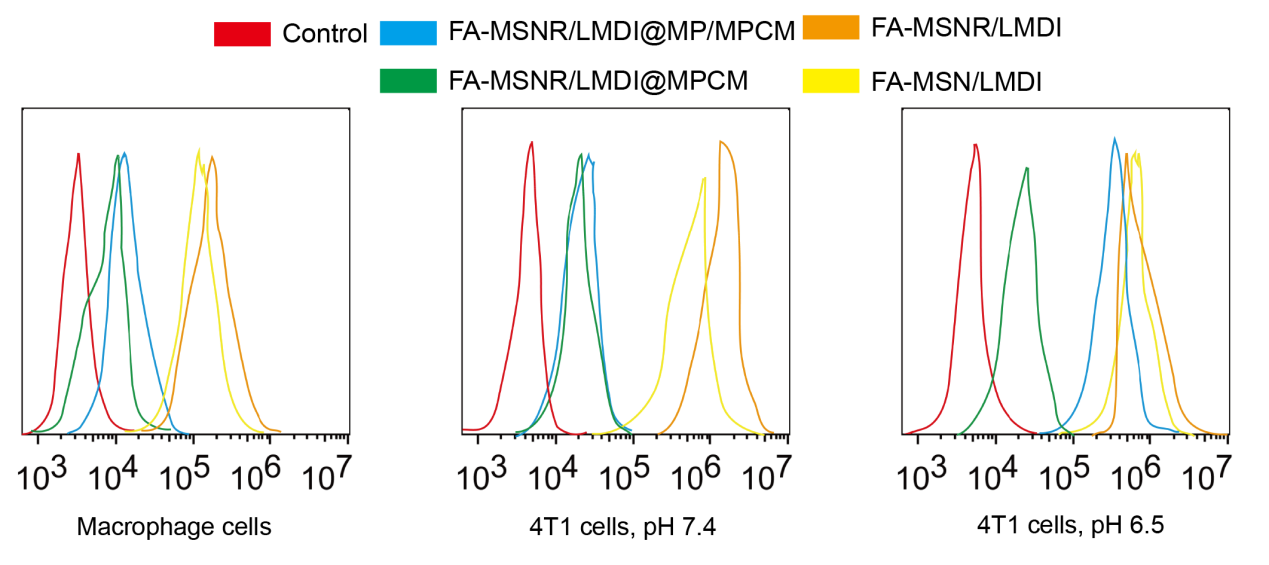


**Fig. S5** Flow cytometry measurements of uptake of the four types of nanocarriers by 4T1 cells and macrophages.


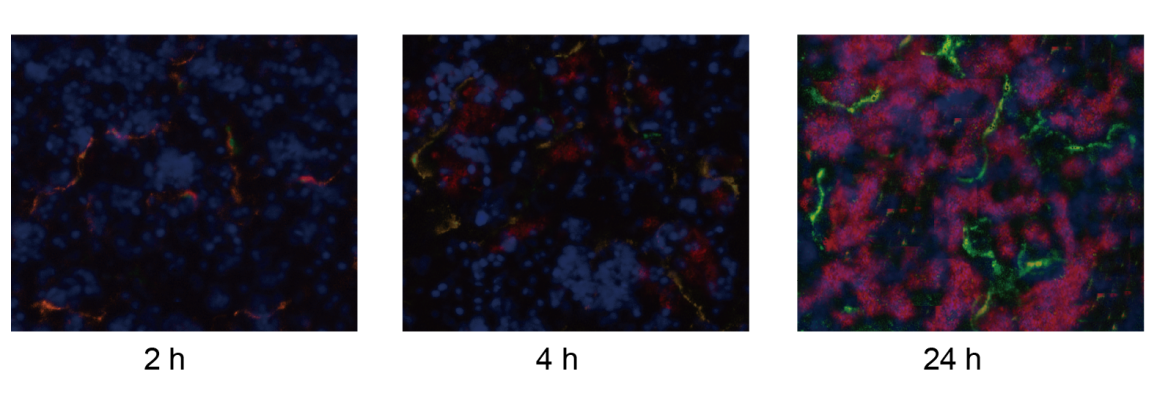


**Fig. S6** Tumor sections of tumor-bearing nude mice 2, 4 and 24 h after injection with the four types of nanocarriers. Blue: DAPI; green: CD34; red: nanocarriers.


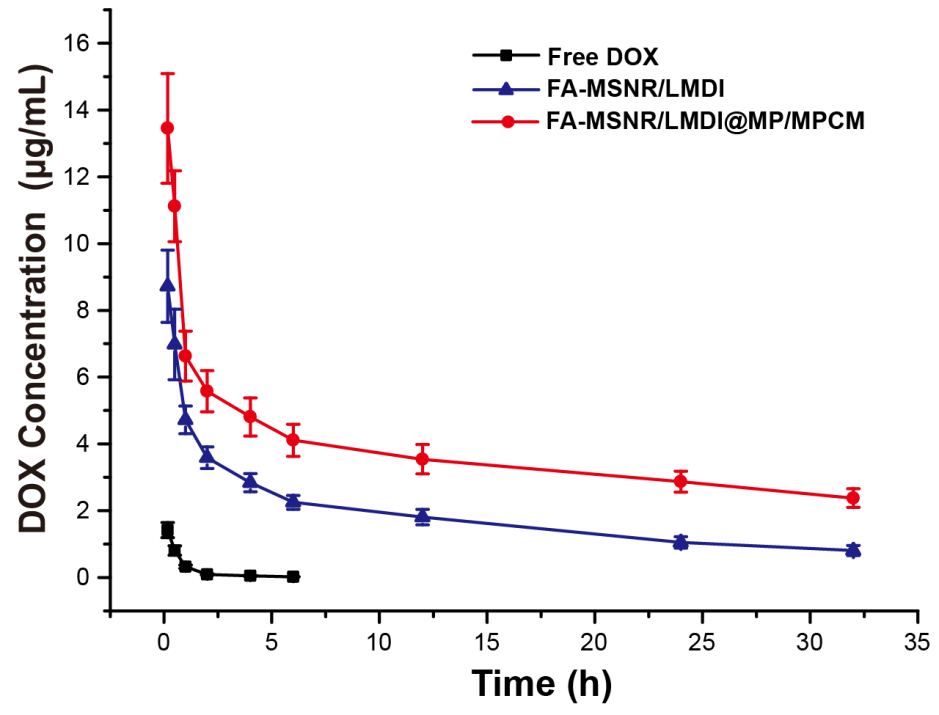


**Fig. S7** Concentration of DOX in plasma at different time after intravenous injection of free DOX, FA-MSNR/LMDI and FA-MSNR/LMDI@MP/MPCM.


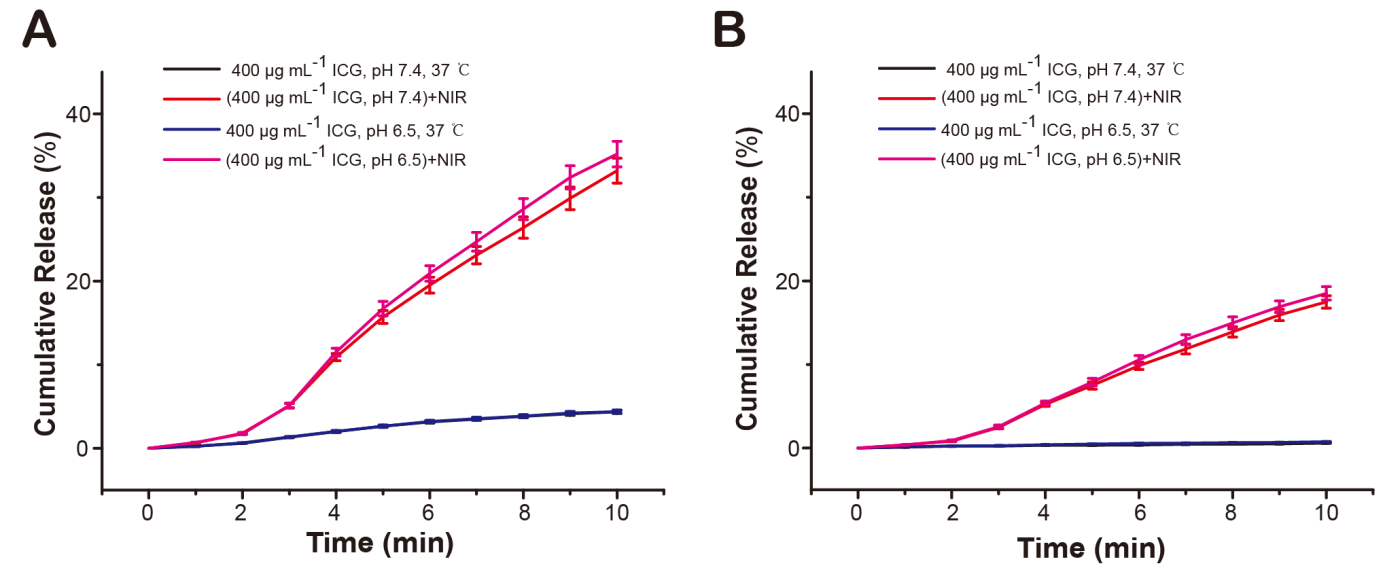


**Fig. S8** Accumulative Dox release curves of FA-MSNR/LMDI (**A**) and FA-MSNR/LMDI@MPCM (**B**) containing 400 μg mL^-1^ ICG at 37 ℃, pH 7.4 or pH 6.5, with or without NIR irradiation for 10 min.

**
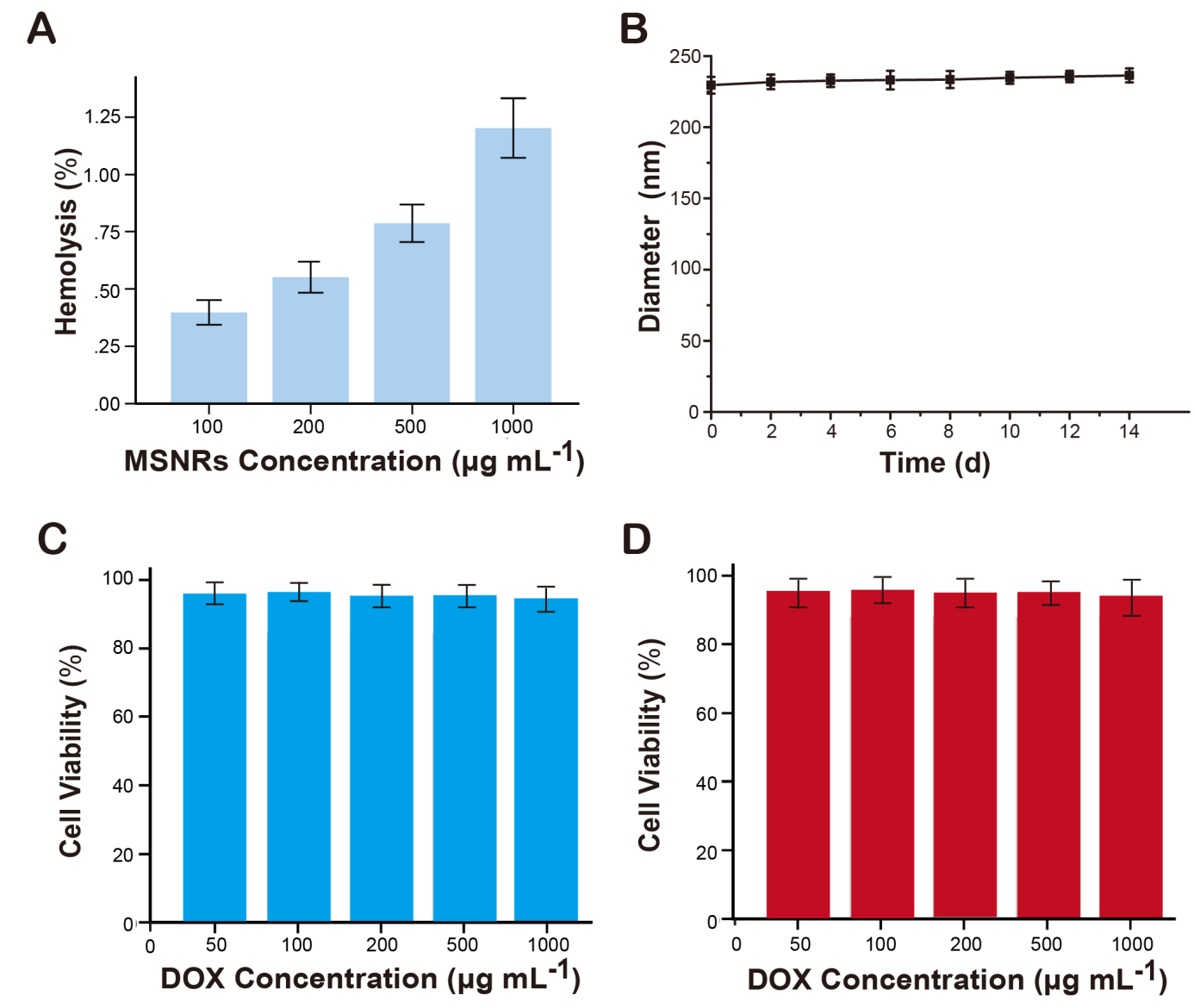
**

**Fig. S9 A** In vitro hematological analysis of MSNRs with various concentrations. **B** Stability analysis of MSNRs in human blood serum. **C** Cytotoxicity of MSNRs with various concentrations on L-02 cells. **D** Cytotoxicity of MSNRs with various concentrations on 293T cells.


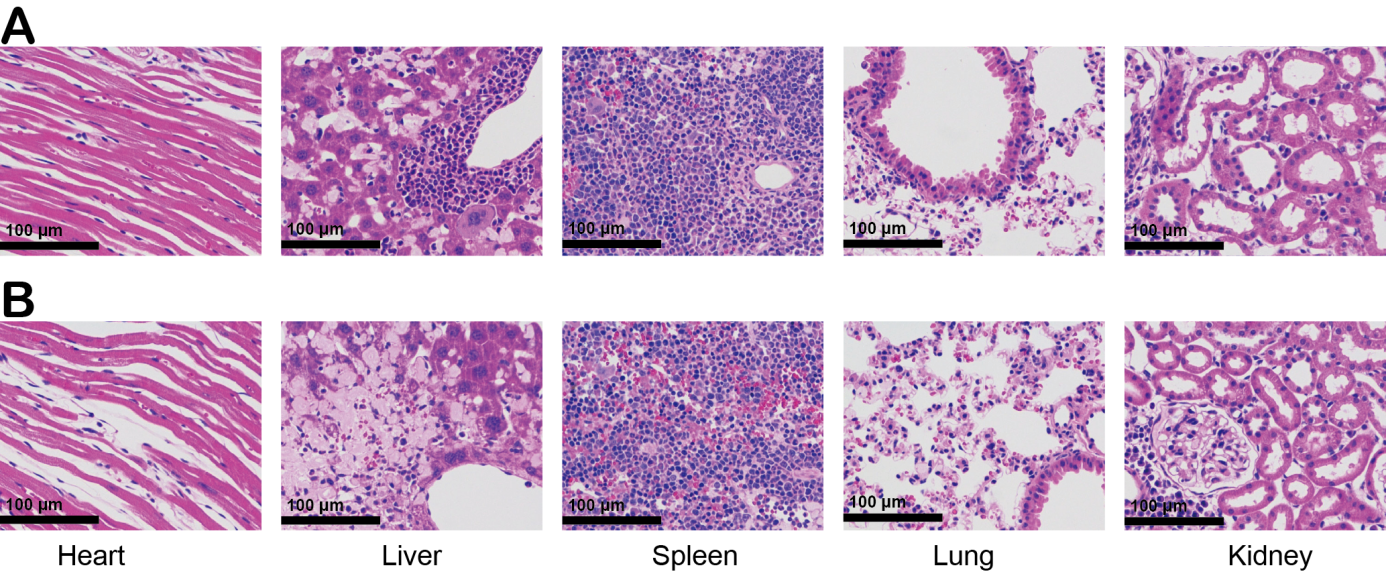


**Fig. S10** Histology stainings of main organs from tumor-bearing nude mice. **A** H&E stainings of the sections of heart, liver, spleen, lung, and kidney from tumor-bearing nude mice after intravenous injection of MSNRs. **B** H&E stainings of the sections of heart, liver, spleen, lung, and kidney from tumor-bearing nude mice after intravenous injection of FA-MSNR/LMDI@MP/MPCM with NIR irradiation.

**
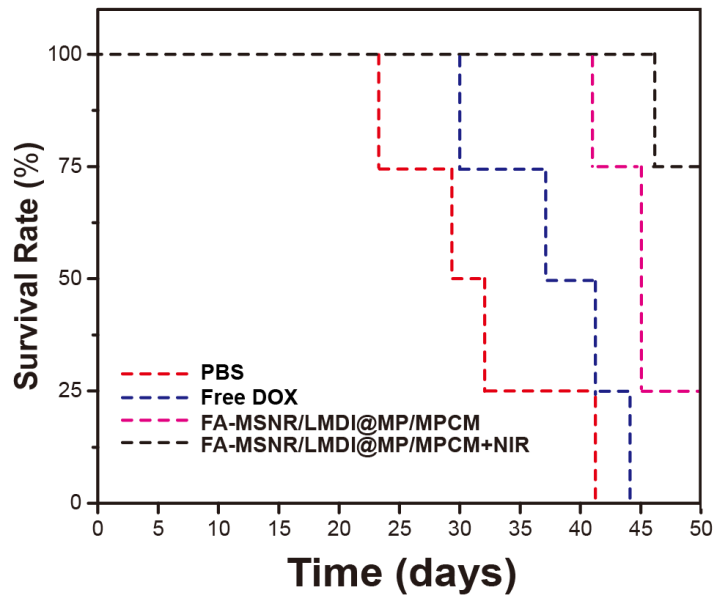
**

**Fig. S11** Percent survival for difffferent treatment groups during 50 days.

**Table. S1** Pharmacokinetic parameters of DOX after intravenously administration of the four types of nanocarriers at the DOX dose of 1 mg kg^-1^ of mouse body weight.

| **Parameters** | **Free DOX** | **FA-MSNR/LMDI**  **@MP** | **FA-MSNR/LMDI**  **@MP/MPCM** |
| --- | --- | --- | --- |
| **T_1/2β_ (h)** | 0.65±0.21 | 23.2±87.24 | 27.84±4.28 |
| **C_max_ (μg/mL)** | 1.41±0.23 | 8.72±1.08 | 13.45±1.64 |
| **Cl (L/h)** | 0.39±0.05 | 0.17±0.03 | 0.05±0.01 |

T_1/2β_: Elimination phase half-life period of medicine.

C_max_: Maximum concentration observed.

Cl: Clearance of medicine.

**Table. S2** Liver and kidney function test

| **Sample** | **ALT** | **AST** | **ALP** | **BUN** | **C_Cr_** |
| --- | --- | --- | --- | --- | --- |
|  | **(U L^-1^)** | **(U L^-1^)** | **(U L^-1^)** | **(mmol L^-1^)** | **(μmol L^-1^)** |
| **Control** | 78.72±5.32 | 187.27±39.28 | 239.2±87.24 | 5.71±1.29 | 23.02±2.21 |
| **MSNRs (1.0 mg kg^-1^)** | 75.52±7.29 | 184.69±26.29 | 244.8±83.97 | 5.24±0.25 | 22.53±3.71 |
| **MSNRs (5.0 mg kg^-1^)** | 77.24±6.41 | 191.97±41.81 | 246.9±88.24 | 6.04±1.94 | 23.39±4.75 |
| **MSNRs (10.0 mg kg^-1^)** | 81.26±5.61 | 186.39±24.97 | 242.6±85.91 | 5.84±0.69 | 21.49±3.27 |
| **FA-MSNR/LMDI@MP/MPCM+NIR** | 82.65±8.61 | 190.71±27.88 | 240.4±82.74 | 5.98±1.09 | 24.19±2.98 |

Notes：Alanine transaminase (ALT)，Aspartate transaminase (AST)，Alkaline phosphatase (ALP)，Blood urea nitrogen (BUN)，Creatinine clearance rate (CCr). Data are shown as mean ± SD.
